# Barriers and enablers in implementing integrated primary health care: A qualitative assessment in West Sumbawa District, Indonesia

**DOI:** 10.1371/journal.pgph.0007238

**Published:** 2026-09-11

**Authors:** Mentari Widiastuti, Sandra Olivia Frans, Siti Nurfadilah Hafala, Shita Listyadewi, Ery Setiawan, Ulfathea Mulyadita, Anooj Pattnaik, Mubasysyir Hasanbasri

**Affiliations:** 1 Center for Health Policy and Management, Faculty of Medicine, Public Health, and Nursing, Universitas Gadjah Mada, Sleman, Daerah Istimewa Yogyakarta, Indonesia; 2 School of Medicine and Psychology, College of Science and Medicine, The Australian National University, Acton, The Australian Capital Territory, Australia; 3 Nossal Institute of Global Health, University of Melbourne, Carlton, Victoria, Australia; 4 Public Health Program, Faculty of Public Health, Universitas Halu Oleo, Kendari, Sulawesi Tenggara, Indonesia; 5 Health Systems Insight, Jakarta, Indonesia; 6 Faculty of Public Health, Universitas Indonesia, Depok, Jawa Barat, Indonesia; 7 Health Systems Insight, Washington, District of Columbia, United States of America; 8 Department of Biostatistics, Epidemiology, and Population Health, Faculty of Medicine, Public Health, and Nursing, Universitas Gadjah Mada, Sleman, Daerah Istimewa Yogyakarta, Indonesia; University of Minnesota, UNITED STATES OF AMERICA

## Abstract

In 2023, Indonesia introduced an integrated primary health care (IPHC) model called *Integrasi Pelayanan Kesehatan Primer* (ILP) as part of the national primary care transformation agenda. ILP seeks to strengthen the network of public primary care providers across sub-district, village, and community levels with a five-cluster model organized around a life-cycle approach, replacing the previously fragmented, program-based model. West Sumbawa District (WSD) in West Nusa Tenggara Province was chosen as a national pilot district by the Ministry of Health (MOH), with ILP implementation expanding from one to all ten public primary care clinics (*Puskesmas*) in 2024. Using a qualitative design, this study aims to evaluate the implementation process, barriers, and facilitators of ILP in WSD. Data were collected through focus group discussions, small group interviews, and individual interviews with health workers, community health workers, and district-level stakeholders across multiple time points between 2023 and 2025. The study found that health system barriers, namely limited human resources availability and capacity across all levels of primary care network, fragmented governance, leadership, and management practices, insufficient financing attributable to limited cross-sectoral support, inadequate physical resources, and a fragmented information system constrained ILP implementation. Despite these barriers, support from local government, community health workers, and the MOH were identified as key enablers. The results demonstrate that establishing IPHC in decentralized settings requires structural and operational redesign as well as recognizing the social, political, and organizational dynamics of the local units where reforms take place. These findings are applicable to broader primary health care (PHC) strengthening initiatives across decentralized contexts.

## Introduction

A primary health care (PHC)-oriented health system is essential for achieving universal health coverage as it offers comprehensive, affordable, and accessible health services to all community members, including health promotion, disease prevention, and rehabilitation services [1]. The significance of PHC has been acknowledged globally, especially since the Declaration of Alma-Ata in 1978 [2]. The World Health Organization and the United Nations Children’s Fund also introduced a PHC theory of change that outlines the pathway to achieve PHC outcomes through integrated health services, empowered individuals, and communities, and multisectoral policy and action [3]. This process depends on support from key strategic and operational factors like political commitment, financing, digital technologies, as well as physical and human resources [3].

Countries worldwide recognize the significance of integrated PHC (IPHC) [4]. Integration is viewed as essential for enhancing health system efficiency through shared visions, resources, and services [5]. Various theories, models, and frameworks have been developed to define and outline the components of integration. Shortell et al. contend that the system or network forming integrated service delivery is shaped by its clinical and fiscal accountability for a specific population [6]. Integration can occur at various levels, including physician-system, functional, and clinical, with the latter being regarded as the most essential for an effective healthcare system [6]. Another model by Singer et al. classifies integration into five types: structural, functional, normative, interpersonal, and process, highlighting the organizational and social characteristics of integration [7] and is an expansion of the model proposed by Shortell et al. [6].

The importance of IPHC has also been acknowledged by the Indonesian government. Since 2001, the country has employed decentralized public health governance, placing the responsibilities in organizing and financing health care under the authority of the district and municipal governments [8]. This approach, however, results in increased inequity due to local heterogeneities [9]. Furthermore, Indonesia has large and complex PHC networks, where there are over 10,000 sub-district primary care clinics (*Puskesmas*), 27,000 village-level auxiliary PHC clinics (*Upaya Pelayanan Kesehatan Desa/Kelurahan*/UPKDK), and 300,000 community health posts (*Posyandu*) spread across 38 provinces and 514 districts and municipalities [10,11]. Basic structural issues, such as fragmented, program-based governance and financing, further impair the performance of the PHC system in meeting the needs of the population [10]. As such, in 2023, Indonesia introduced an IPHC model called *Integrasi Pelayanan Kesehatan Primer* (ILP) as part of the national primary care transformation initiative.

**Fig 1:**
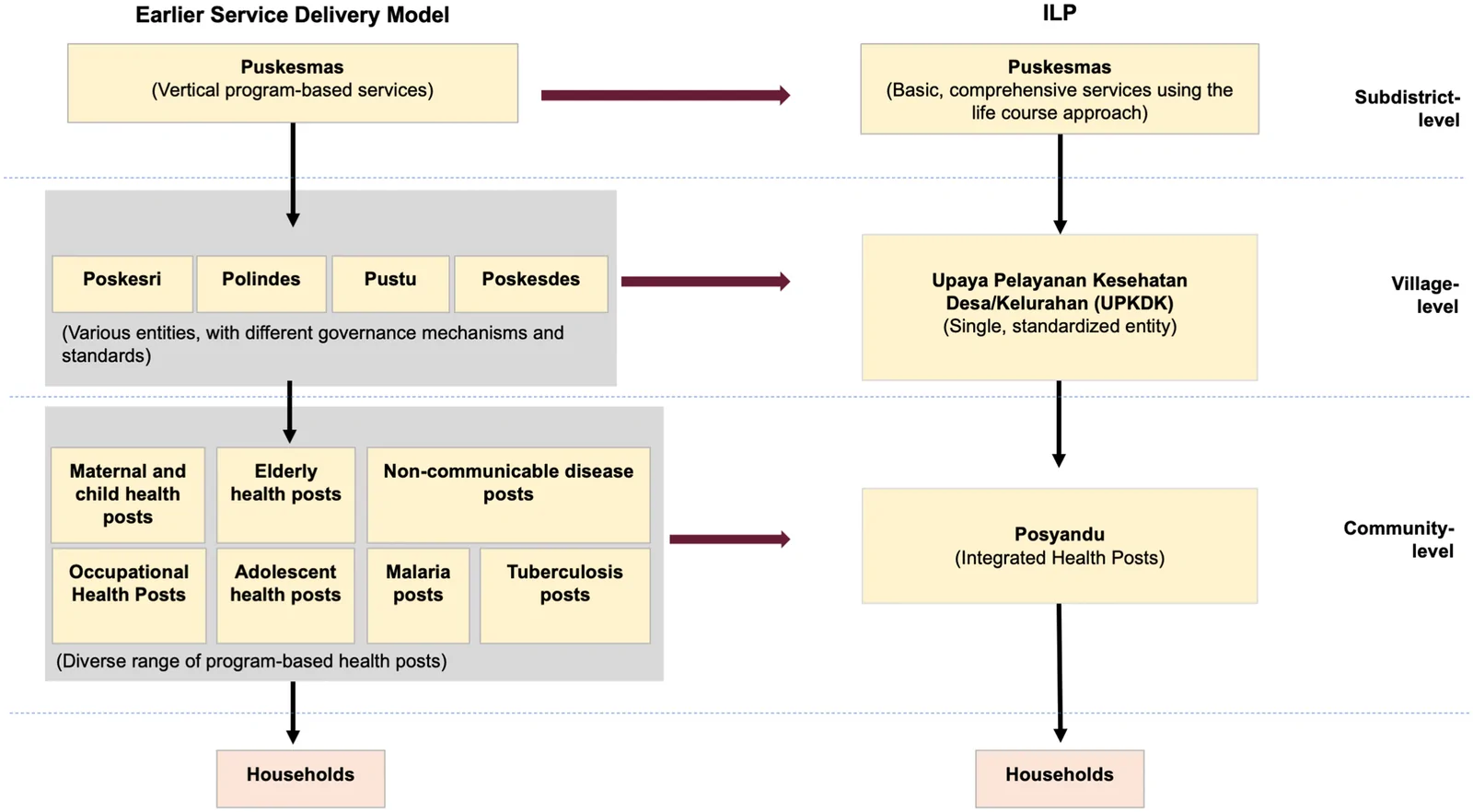
The structure of ILP versus the earlier service model, adapted from Manurung et al. [10]. While the earlier PHC service model consisted of a diverse array of village and community-level facilities, IPHC employed a three-tiered structure, comprising of *Puskesmas* as the main coordinator at the subdistrict level, UPKDK as the village-level facility, and integrated health posts called *Posyandu* at the community level. The aim is to reduce segmentation, enabling patients to receive coordinated and comprehensive care across the system.

With an aim to reorganize PHC to be integrated, comprehensive, and accessible, ILP involves three reform elements, namely structural, service, and performance reforms [10], which align with the frameworks introduced by Shortell et al. [6] and Singer et al. [7]. The structural reform focuses on strengthening the network of primary care providers at sub-district, village, and community levels, with *Puskesmas* acting as the coordinator for UPKDK and *Posyandu* [10,12] (Fig 1).

The reform also requires that at least two health workers and two community health workers (CHWs) must be assigned at a UPKDK clinic, while a *Posyandu* facility must have at least five CHWs [12]. The service reform aims to offer more comprehensive services by shifting from fragmented, program-based delivery to a model organized around five clusters based on the life-cycle approach, emphasizing prevention and early detection [10]. The life cycle approach acknowledges that health is dynamic and affected by various determinants throughout life [13]. Within the ILP framework, this is reflected in two of the five clusters, namely maternal and child health, as well as productive age and elderly health [10]. Lastly, the performance reform seeks to embed routine performance monitoring and multi-level data-driven decision-making via a village local area monitoring (LAM) dashboard [10]. ILP was piloted in nine districts, one of which was West Sumbawa District (WSD) in West Nusa Tenggara Province [14].

There is a notable gap within the existing literature that examines IPHC as a system-wide reform in decentralized contexts. Many studies on IPHC focus on integrating specific services, such as non-communicable diseases [15–19], human immunodeficiency virus infection [20–22], and tuberculosis [23–25], into the PHC. Most of these studies also offer limited insights on how decentralization and broader health system factors shape the implementation. To fill gaps in the existing knowledge, our study adopts a comprehensive system-wide approach to examine how an IPHC model was implemented at the district level, where reforms are enacted within the Indonesian decentralized system. We used the case of WSD as the national pilot site of ILP and captured the perspectives of district actors involved in ILP, namely health workers and CHWs delivering frontline services, the District Health Office (DHO) managing health affairs at sub-national level, and the District Village Community Empowerment Office (DVCEO) overseeing village development, to document practices, challenges, and lessons learned from ILP implementation. This paper has two main objectives, namely, to understand participants’ perspectives on ILP as a new, integrated service model, and to identify barriers and facilitators affecting ILP implementation and scaling-up within the pilot district.

## Materials and methods

### Ethics statement

The study received ethics approval from the Internal Review Board of the Faculty of Medicine, Public Health, and Nursing at Universitas Gadjah Mada, Indonesia (Approval Number: KE/FK/1206/EC/2023, KE/FK/1322/EC/2024, KE/FK/1275/EC/2025). Participants provided formal written consent, both in printed and digital formats, for each online or face-to-face data collection. Personally identifiable information was removed from the transcripts to safeguard participants’ anonymity and confidentiality. Data privacy measures were implemented.

### Study design

The study employed a qualitative design using thematic analysis with a realist paradigm. As described by Braun and Clarke, a realist paradigm allows a straightforward interpretation of meaning based on participants’ statements [26]. The study was performed and reported in accordance with the Consolidated Criteria for Reporting Qualitative Research (COREQ) framework [27] (S1 File).

### Study settings

This study was conducted in WSD, West Nusa Tenggara Province, Indonesia. As of 2025, the district has a population of 155,540 people [28]. Since 2016, WSD has implemented a program called *Gotong Royong* [29]*.* The program aims to improve the accessibility and responsiveness of public services, including those in the health sector [29]. Since 2021, with the establishment of community-level integrated posts called *Posyandu Gotong Royong* [30]*,* there has been an opportunity for the program to bring health services closer to communities, to as well as integrate health with other social determinants. This approach was deemed aligned with ILP’s aim to provide comprehensive and integrated health services, leading to WSD being selected as a national ILP pilot site in 2022 [31,32]. When the pilot commenced in 2022, the district had 9 *Puskesmas* clinics, 97 UPKDK clinics (including 30 clinics owned by *Puskesmas* called *Pustu* and 67 clinics owned by the village government called *Poskesdes*), and 229 *Posyandu* facilities [33]. The pilot was conducted in one *Puskesmas* clinic and its two associated UPKDK clinics. The pilot *Puskesmas* and UPKDK clinics received practical technical assistance from the MOH and the DHO and were visited by the Minister of Health for direct supervision and monitoring [34]. In 2024, one *Puskesmas* clinic underwent a division, resulting in a total of ten *Puskesmas* clinics in the district [35]. As of 2024, all *Puskesmas* clinics had met the MOH’s ILP criteria, namely 1) employing the ILP cluster-based organizational structure, formalized by a Head of *Puskesmas* Decree, 2) having standard operating procedures for service delivery in each cluster, 3) having at least one UPKDK clinic providing cluster-based services and community empowerment activities, and 4) having at least one *Posyandu* facility providing integrated services and home visits [36].

### Sampling

**Fig 2:**
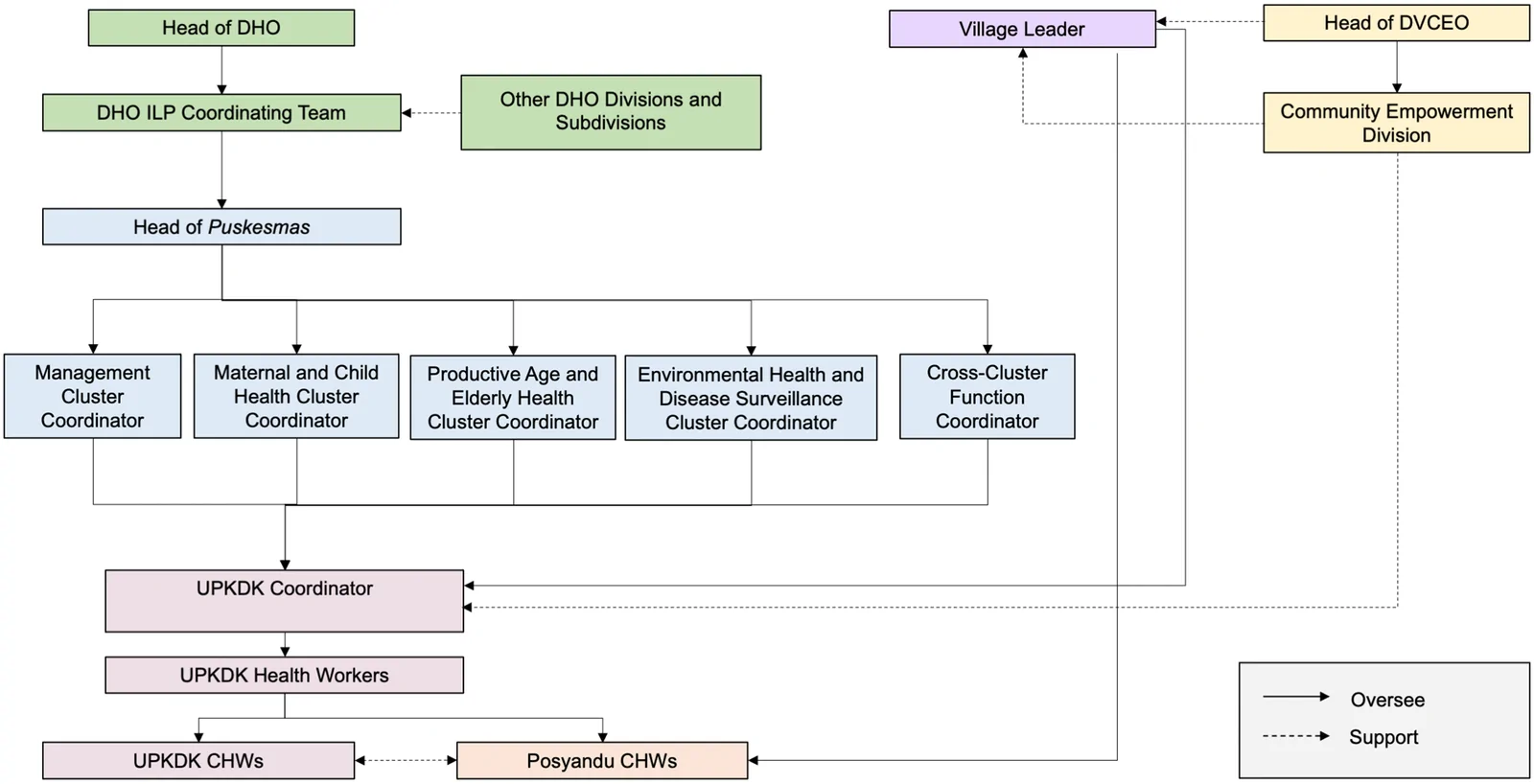
The leadership structure of ILP in WSD. The DHO holds the primary responsibility to coordinate and oversee the ILP network, comprising *Puskesmas*, UPKDK, and *Posyandu.* The DVCEO also plays pivotal roles in the ILP structure by supporting village leaders in financing and overseeing UPKDK and *Posyandu*.

Purposive sampling was employed to recruit our participants, including the heads of *Puskesmas* clinics, health workers at *Puskesmas* and UPKDK clinics, CHWs at UPKDK clinics and *Posyandu* facilities, as well as representatives from the DHO and the DVCEO who had at least one year of experience working in their respective units, ensuring they had adequate exposure and understanding of the health services provided. We included both pilot and non-pilot facilities across urban and rural areas, following discussions with the DHO. The CHWs were selected in consultation with *Puskesmas* and UPKDK, subject to meeting the inclusion criteria. The participants chosen represented all key stakeholders involved in the district-level governance of ILP (Fig 2). No eligible participants declined to take part in the study.

### Data collection

Our primary data collection method was focus group discussions (FGDs) comprising six to ten participants in each group to explore participants’ experiences and perspectives regarding ILP implementation in WSD from November 2023 to May 2025. Additionally, we conducted individual interviews (involving one participant in each session) and small group interviews (involving two to five participants in each session) to gather unique, more in-depth insights from participants. This categorization of qualitative data collection activities follows the examples of Dahlgren et al. [37] and Carino et al. [38]. Each FGD took 90–120 minutes, whereas the duration of each interview was around 60 minutes. The guide employed open-ended questions related to participants’ professional background and the study objectives (S2 File). The guide was tested for its clarity and relevance through discussions among authors and consultation with a senior expert in PHC in Indonesia.

Overall, our participants included representatives from the DHO, DVCEO, 1 pilot *Puskesmas* clinic, 6 non-pilot *Puskesmas* clinics, 1 pilot UPKDK clinic, and 8 non-pilot UPKDK clinics. Relationships with the DHO and DVCEO were established through the facilitation of the MOH’s PHC consortium (co-led by Health Systems Insight). The DHO further facilitated our communication with the *Puskesmas* and UPKDK. Prior to the data collection, participants were informed about the study objectives and procedures in online and face-to-face formal meetings. Since having institutional endorsement is considered culturally appropriate for participant recruitment in the study site, we also distributed institutional letters through the DHO to formally invite government officials, healthcare providers, and CHWs to participate in the study.

All interviews and group discussions were led by the first, second, and third authors, who are female Indonesian researchers with a master’s degree in public health, trained in qualitative research, and working in a university-based research institute. They have at least five years of experience in public health research and professional interests in health system and policy topics. The first and second authors also have prior experience working as a pharmacist and a clinician, respectively, in primary care settings. All data collection activities were conducted in formal Indonesian language, which is the standard practice when engaging with government officials and health care providers.

Data collection was conducted across five key time points from 2023 to 2025 to capture the changing context of ILP implementation in WSD (Table 1). The first round of data collection took place in November 2023, during the early phase of ILP rollout, when only one *Puskesmas* clinic, that is the pilot clinic, adopted the model following the issuance of the Minister of Health Decree Number 2015 of 2023 on ILP [12]. In this phase, we conducted an online FGD with DHO representatives. The second and third rounds occurred in February and March 2024, respectively. These rounds happened approximately six months after the national ILP launch and 18 months after the pilot started, with the pilot *Puskesmas* clinic being the only one meeting the MOH’s criteria for ILP. The second round involved an online FGD with two non-pilot *Puskesmas*, while the third comprised face-to-face small group interviews and FGDs with pilot *Puskesmas* clinic, pilot and non-pilot UPKDK clinics, *Posyandu* facilities, and the DHO. The fourth round took place in September 2024, when a new *Puskesmas* clinic was established and all other *Puskesmas* clinics had been declared to meet the MOH’s ILP criteria. This round included face-to-face interviews (both individual and small group) and FGDs with pilot and non-pilot *Puskesmas* and UPKDK clinics, the DVCEO, and the DHO. The fifth and final round was conducted in May 2025, around three years after the pilot’s inception and amidst national and local leadership transitions. This phase involved face-to-face FGDs with pilot and non-pilot *Puskesmas* clinics, UPKDK clinics, and the DHO. During this round, new national and local policies were developed, including the issuance of the Minister of Health Regulation Number 19 of 2024 on *Puskesmas*, which revoked the earlier Minister of Health Regulation Number 43 of 2019 on *Puskesmas* [39]. The introduction of the new *Puskesmas* regulation is significant as it explicitly states that PHC is conducted using a life cycle-based integrated PHC model, as opposed to the program-based approach outlined in the previous regulation, thus aligning with ILP [39]. Another contextual change was the initiation of the national free health screening program for all age groups as part of the new president’s key health innovations [40], which complements the initiative to deliver comprehensive life-cycle based health screenings in ILP. The recruitment start and end dates for each round of data collection are as follows: 5–8 November 2023, 19–20 February 2024, 29 February–2 March 2024, 18–20 September 2024, and 16–18 May 2025.

**Table 1:** List of data collection activities.

Data Collection Round	Data Collection Activity	Mode of Delivery	Participant	Number of Participants
November 2023	FGD	Online	DHO	9
February 2024	FGD	Online	Non-pilot *Puskesmas* 04 and 05 (health workers)	11
March 2024	FGD	Face-to-face	DHO	6
	FGD	Face-to-face	Pilot *Puskesmas* (health workers)	8
	Small-group interview	Online	Non-pilot UPKDK 06, 07, and 08 (health workers)	5
	Small-group interview	Face-to-face	Pilot UPKDK (health workers and CHWs)	4
	Small-group interview	Face-to-face	*Posyandu* CHWs under pilot UPKDK	4
	Small-group interview	Face-to-face	Non-pilot UPKDK 01 (health workers and CHWs)	4
	Small-group interview	Face-to-face	*Posyandu* CHWs under non-pilot UPKDK 01	4
September 2024	FGD	Face-to-face	DHO	7
	FGD	Face-to-face	Non-pilot *Puskesmas* 01 (health workers)	7
	FGD	Face-to-face	Non-pilot *Puskesmas* 02 (health workers)	7
	FGD	Face-to-face	Non-pilot *Puskesmas* 03 (health workers)	7
	FGD	Face-to-face	Non-pilot UPKDK 02 (health workers and CHWs)	7
	FGD	Face-to-face	Non-pilot UPKDK 04 (health workers and CHWs)	6
	Small-group interview	Face-to-face	DVCEO	2
	Small-group interview	Face-to-face	Pilot *Puskesmas* (health workers)	3
	Small-group interview	Face-to-face	Pilot UPKDK (health workers and CHWs)	4
	Small-group interview	Face-to-face	Non-pilot UPKDK 03 (health workers and CHWs)	4
	Individual interview	Face-to-face	Non-pilot *Puskesmas* 06 (health workers)	1
May 2025	FGD	Face-to-face	DHO	8
	FGD	Face-to-face	Pilot *Puskesmas* (health workers)	7
	FGD	Face-to-face	Non-pilot *Puskesmas* 01 (health workers)	8
	FGD	Face-to-face	Non-pilot *Puskesmas* 02 (health workers)	2
	FGD	Face-to-face	Non-pilot *Puskesmas* 05 (health workers)	5
	FGD	Face-to-face	Non-pilot *Puskesmas* 06 (health workers)	6
	FGD	Face-to-face	Pilot UPKDK (health workers and CHWs)	6
	FGD	Face-to-face	Non-pilot UPKDK 04 (health workers and CHWs)	6
	FGD	Face-to-face	Non-pilot UPKDK 05 (health workers and CHWs)	8

Online data collection activities were conducted using an online meeting platform, while in-person data collection activities were conducted in participants’ offices. Only the researchers and participants were present in the room when data collection took place.

### Data analysis

All interviews and FGDs were audio recorded, transcribed verbatim, and manually coded using a structured Microsoft Excel spreadsheet. All transcripts were fully anonymized, and participants were assigned generic labels to ensure anonymity. While we did not return transcript to participants for comments or correction, preliminary results were presented to the DHO in online routine meetings, during which the DHO would provide their confirmation, feedback, and additional contexts to the results presented. Comments and feedback from the DHO were used to contextualize our findings but did not influence the thematic analysis process. The development of final themes remained grounded in the perspective of participants to preserve the independence and authenticity of their voices.

A thematic analysis was conducted for all qualitative data following the structured six phase framework outlined by Braun and Clarke [26], ensuring both methodological rigor and analytical depth. The analysis was conducted using a semantic approach, with the interpretation of broader meanings and implications directly based on participants’ description of their views and experiences [26]. The analytical stages included data familiarization, initial coding, development of themes and categories, review of codes and emergent subthemes, interpretation and presentation of findings, and final writing. During the familiarization stage, the study team engaged in repeated readings of the transcripts to achieve deep immersion in the dataset. During initial coding, all transcripts were coded using a shared codebook and labelled with pilot and non-pilot. Coded data were then compared across pilot and non-pilot PHCs during interpretation. During theme development, transcripts from both groups were analyzed together to identify themes that reflected shared meanings across implementation contexts. Although the number of pilot and non-pilot facilities was not equal, non-pilot facilities had been gradually exposed to ILP implementation throughout the data collection period. As such, participants’ accounts reflected different stages of ILP implementation rather than entirely distinct contexts of pilot and non-pilot facilities. Themes were developed based on the interpretation of transcripts using a semantic approach. We further categorized the themes into three major groups, namely implementation description, barriers, and facilitators. These themes were developed based on the updated United Kingdom Medical Research Council (MRC)/National Institute for Health and Care Research (NIHR) Framework for Complex Interventions, which emphasized inquiring beyond whether an intervention achieves its desired results, but involves asking a range of questions, including how the intervention works and how it relates to the context in which it is embedded [41].

Coding was performed independently by the first author to identify meaningful data segments. The second and third authors also coded the transcripts to enhance reliability. All authors then met to discuss code refinement and develop initial themes. These themes were then systematically reviewed across the entire dataset to assess their coherence, distinctiveness, and alignment with participants’ experiences. Field notes taken throughout all data collection rounds were also checked if there were discrepancies during the data interpretation and analysis process. Throughout the analytic process, comprehensive documentation including analytic notes and reflexive memos was maintained to record decisions and improve transparency and overall study rigor. To verify saturation, the first, second, and third authors continuously examined themes generated across all data collection rounds. In the final round (May 2025), no novel themes were generated and the data fit within the existing thematic structure.

## Results

Throughout the data collection phases, our study involved a total of 138 individuals representing the DHO, DVCEO, one pilot *Puskesmas*, six non-pilot *Puskesmas*, one pilot UPKDK, and eight non-pilot UPKDK involved in FGDs and in-depth interviews (Table 2). Some participants took part in more than one data collection round.

**Table 2:** Total number of participants throughout the study.

Organization	n	%
DHO	24	17.4
DVCEO	2	1.4
Pilot *Puskesmas*	12	8.7
Non-pilot *Puskesmas* 01	13	9.4
Non-pilot *Puskesmas* 02	9	6.5
Non-pilot *Puskesmas* 03	7	5.1
Non-pilot *Puskesmas* 04	6	4.3
Non-pilot *Puskesmas* 05	9	6.5
Non-pilot *Puskesmas* 06	6	4.3
Pilot UPKDK	11	8.0
Non-pilot UPKDK 01	8	5.8
Non-pilot UPKDK 02	7	5.1
Non-pilot UPKDK 03	4	2.9
Non-pilot UPKDK 04	7	5.1
Non-pilot UPKDK 05	8	5.8
Non-pilot UPKDK 06	2	1.4
Non-pilot UPKDK 07	1	0.7
Non-pilot UPKDK 08	2	1.4
**Total**	**138**	**100.0**

As shown in Table 3, the study included between 9 and 56 participants in each round. Most of these participants were women (54.5%-85.7%). Some participants, particularly from the DHO, pilot *Puskesmas*, pilot UPKDK, non-pilot *Puskesmas* 1, non-pilot *Puskesmas* 2, non-pilot *Puskesmas* 6, and non-pilot UPKDK 4, participated more than once.

**Table 3:** Characteristics of participants.

Characteristics		November 2023 (n = 9)		February 2024 (n = 11)		March 2024 (n = 35)		September 2024 (n = 55)		May 2025 (n = 56)	
		n	%	n	%	n	%	n	%	n	%
**Gender**	Women	6	66.7	6	54.5	30	85.7	40	72.7	45	80.4
	Men	3	33.3	5	45.5	5	14.3	15	27.3	11	19.6
**Institution**	DHO	9	100.0	0	0.0	6	17.1	7	13.0	8	14.3
	DVCEO	0	0.0	0	0.0	0	0.0	2	3.6	0	0.0
	Pilot *Puskesmas*	0	0.0	0	0.0	8	22.9	3	5.5	7	12.5
	Non-pilot *Puskesmas* 01	0	0.0	0	0.0	0	0.0	7	12.7	8	14.3
	Non-pilot *Puskesmas* 02	0	0.0	0	0.0	0	0.0	7	12.7	2	3.6
	Non-pilot *Puskesmas* 03	0	0.0	0	0.0	0	0.0	7	12.7	0	0.0
	Non-pilot *Puskesmas* 04	0	0.0	6	54.5	0	0.0	0	0.0	0	0.0
	Non-pilot *Puskesmas* 05	0	0.0	5	45.5	0	0.0	0	0.0	5	8.9
	Non-pilot *Puskesmas* 06	0	0.0	0	0.0	0	0.0	1	1.8	6	10.7
	Pilot UPKDK	0	0.0	0	0.0	8	22.9	4	7.3	6	10.7
	Non-pilot UPKDK 01	0	0.0	0	0.0	8	22.9	0	0.0	0	0.0
	Non-pilot UPKDK 02	0	0.0	0	0.0	0	0.0	7	12.7	0	0.0
	Non-pilot UPKDK 03	0	0.0	0	0.0	0	0.0	4	7.3	0	0.0
	Non-pilot UPKDK 04	0	0.0	0	0.0	0	0.0	6	10.9	6	10.7
	Non-pilot UPKDK 05	0	0.0	0	0.0	0	0.0	0	0.0	8	14.3
	Non-pilot UPKDK 06	0	0.0	0	0.0	2	5.7	0	0.0	0	0.0
	Non-pilot UPKDK 07	0	0.0	0	0.0	1	2.9	0	0.0	0	0.0
	Non-pilot UPKDK 08	0	0.0	0	0.0	2	5.7	0	0.0	0	0.0

We identified three main themes related to the ILP rollout, including its description, key barriers, and key facilitators of ILP implementation based on participants’ perceptions (S3 File).

### Description of ILP implementation

#### Familiarity with the ILP concept.

In general, there were no perceived substantial changes to health care practices at the *Puskesmas* and *Posyandu* level. The concept of ILP was not considered entirely new by our health workers and CHWs participants. *Puskesmas* staff stated that service delivery in the ILP model was essentially similar to the previous model, with the main difference being the grouping of services based on life-cycle clusters.

*“We were not surprised by the ILP service in West Sumbawa District. It is almost the same, just grouped by age. Otherwise, there are no issues, except that those under 15 with families are included in cluster three.”*

*(Health worker, Non-pilot Puskesmas 05, February 2024)*

In contrast to findings at the *Puskesmas* level, changes at the village level, signified by the presence of UPKDK, were regarded as vital in meeting the community’s needs for more accessible health care services. With the implementation of ILP, UPKDK is strengthened and equipped with health workers stationed there, as illustrated in the following quote.

*“Initially, it [UPKDK] was a Poskesdes. People prefer to visit the Poskesdes rather than Puskesmas because it is closer. The Poskesdes was built in 2012 and was transformed into Posyandu Prima [the earlier nomenclature before being changed into UPKDK] in 2023, because of ILP. We have one midwife and one nurse, with also the CHWs.”*

*(Health worker, Non-pilot UPKDK 07, November 2023)*

District stakeholders regarded the *Posyandu Gotong Royong* program as a vital catalyst for ILP. The program had been implemented by the district government before the launch of ILP. *Gotong Royong* is an Indonesian term that describes collaborative and collective responsibility. *Posyandu Gotong Royong* was designed to meet community needs for public services across all sectors, as well as to strengthen community empowerment and development.

*“It was previously known as Posyandu Keluarga, then as Posyandu Gotong Royong, and is now referred to as Posyandu Prima. In the program, many entities participate in implementing health, including CHWs. We also have Gotong Royong agents, and we provide immunization services, health promotion, nutrition, pregnant women, and environmental health. All is provided at Posyandu Prima.”*

*(DHO Staff, November 2023)*

### Adoption process of ILP

Within the pilot district, the extent of ILP adoption between pilot and non-pilot facilities varied. For instance, some non-pilot UPKDK clinics struggled in organizing their services around the life-cycle approach. The differences happened because capacity building and technical assistance from the DHO to non-pilot *Puskesmas*, UPKDK, and *Posyandu* were limited. Thus, facility managers at non-pilot sites attempted to learn independently to implement ILP by visiting the pilot *Puskesmas* and UPKDK. Due to the lack of preparation, many non-pilot facilities remained unclear about ILP and how to implement it. Furthermore, gaps in the knowledge transfer from *Puskesmas* to UPKDK and *Posyandu* led to confusion among the lower-level facilities.

*“[ILP is still progressing] slowly because this is the first year we... no, actually, it’s been happening since last year. I only got the orientation [from Puskesmas] in December [2024]. Even then, it was just an orientation and was very difficult to understand. Although we were progressing, it was going slowly. Until the three of us visited the pilot Puskesmas.”*

*(Health worker, Non-pilot UPKDK 05, May 2025)*

While the levels of ILP adoption varied, this study identified a shared experience among pilot and non-pilot facilities in terms of service reform. The reform emphasizes on a shift from a program and service category-based model to a life cycle-based approach organized into five functional clusters (management, maternal and child health services, adult and elderly services, communicable disease control, and cross-cluster). Through ILP, health workers were expected to perform a wider range of health screenings. Both pilot and non-pilot *Puskesmas* found it challenging to adapt to the new service requirements due to their limited skills and capacities.

*“Science keeps developing, Ma’am, particularly in terms of screening. We have never been specifically trained to conduct health screening for children. There are 39 screening items that we have to perform. We cannot do all of them at once. This is also related to the availability of equipment. Our equipment is limited, but some screening items, such as OB-GYN [obstetrics and gynecology] and lung screening, require advanced equipment. It also applies to thalassemia screening. We do not have the equipment. As such, we start from what we can do, based on our capacity and the availability of equipment.”*

*(Health worker, Pilot Puskesmas, September 2024)*

The expanded screening requirements also led to longer waiting times. This was especially worrying in *Posyandu*, which often lacked the infrastructure to allow patients to wait comfortably.

*“There was a scheduled check-up, but even though someone arrived late, they wanted to be examined first, which is why the Posyandu was [very crowded] like a market.”*

*(Health worker, Non-pilot UPKDK 01, September 2024)*

UPKDK health workers also perceived an increased workload and longer working hours after ILP because they were stationed close to the community, and their roles were expanded. Before ILP, UPKDK focused on promotive and preventive services, especially around maternal and child health. Since ILP was implemented, they had been expected to provide comprehensive services beyond maternal and child health, which used to be primarily delivered by *Puskesmas*. Additionally, UPKDK health workers had to deliver outpatient services five days a week, conduct home visits, and refer emergency cases to *Puskesmas*.

*“There are no [fixed] working hours, Ma’am, because there are no Puskesmas nearby. I am the midwife responsible for managing this Poskesdes. We operate from Monday to Friday, from 8 a.m. to 4 p.m., as we live in the village health post. We provide a 24-hour service, but not specifically for emergencies; we only assist patients who are about to give birth.”*

*(Health worker, Non-pilot UPKDK 06, November 2023)*

### Key Barriers of ILP implementation

#### Human resources.

Participants mentioned challenges in human resources availability across all levels of primary care providers. A shortage of general physicians, dentists, medical technologists, and support staff, such as ambulance drivers, was identified at the *Puskesmas* level throughout the data collection periods. To meet the workforce needs at UPKDK, some *Puskesmas* also allocated their nurses and midwives to UPKDK, causing further shortages.

*“For example, there should be four people on duty in cluster five because we have so many patients. Now, there are three [people] because we must assign one person to the village clinic.”*

*(Health worker, Non-pilot Puskesmas 03, September 2024)*

Participants also reported difficulties in staffing UPKDK with at least two health practitioners and two CHWs, as required by the MOH. Working at the UPKDK, according to participants, was not seen as attractive among health professional graduates. Staff retention was also a concern, with a high turnover of CHWs linked to limited incentives or changes in village leadership.

*“Regarding these CHWs and the current political dynamics... let us consider it. Just yesterday, we, health care professionals at the Puskesmas, trained new cadres. We did this because the old CHWs who had been trained, due to political changes, I suppose, were replaced. The old CHWs were replaced by new ones. Naturally, we need to train these new CHWs. That is what I mean by political changes. They lead to the assignment of new CHWs, who are not yet familiar with health matters.”*

*(Health worker, Non-pilot Puskesmas 06, May 2025)*

Challenges in recruiting and retaining *Posyandu* CHWs were consistently highlighted throughout the study. Most CHWs were mature women who had to balance family responsibilities alongside their duties at *Posyandu*. The lack of interest among men and young people in becoming CHWs, coupled with insufficient incentives (approximately IDR 200,000 or USD 12 per month), was also mentioned.

*“[Men do not want to be CHWs because] They’re busy, they are not interested in [a position with] a small salary, I think. We [women], on the other hand, do not mind the small incentives. At least [the incentive] is enough for buying soap.”*

*(Posyandu CHW, Pilot UPKDK, March 2024)*

Insufficient workforce quality was also cited as a barrier to implementing ILP. The ILP’s cluster-based system required health workers to deliver comprehensive services tailored to patients’ age groups. This was markedly different from the earlier program-based system, which assigned health workers to handle specific health conditions. Several health workers at *Puskesmas* and UPKDK struggled to adapt to the transition, citing inadequate training and preparation as the primary reasons.

*“Yes, in general, as I just explained, the technical guidelines of ILP are already available. However, in practice, we are still trying to figure it out ourselves based on our own understanding of the DHO’s explanation.”*

*(Health worker, Non-pilot Puskesmas 02, September 2024)*

*Posyandu* CHW participants also reported not being familiar with the increasingly digitalized system, as required by the MOH. For instance, the ILP guidelines mandated the use of a mobile phone application named ASIK for *Posyandu* CHWs. However, many CHWs never enrolled in higher education and were rarely exposed to the latest technology. As such, they reported difficulties in using data recording and reporting applications.

*“We already have five CHWs in each Posyandu. However, there have been many upgrades to the data entry application, and not all cadres are well-educated. You know, some are elementary school graduates [and therefore need to learn to use communication devices].”*

*(Health worker, Pilot UPKDK, March 2024)*

#### Information systems.

The implementation of ILP has also resulted in an increase in the number of data reporting applications required to be completed by health care workers at *Puskesmas*. This created an additional administrative burden because each cluster often used a different reporting system, even though the requested data was similar, leading to confusion at the implementation level.

*“We are frankly confused; the Minister of Health Regulation on implementing ILP has been put into practice. But on the one hand, policymakers above it, at district, provincial levels, or I do not know what ministry, are still asking us to complete the old form. Why don’t we have a unified vision? There should be a comprehensive 1-2-3-4 cluster form so that we do not have multiple applications to fill out, even if the data is the same”*

*(Health worker, Non-pilot Puskesmas 05, May 2025)*

Health workers and CHWs needed to enter data into multiple applications, such as E-Puskesmas (for clinical services and management system at *Puskesmas*), SIGIZI (for nutrition surveillance), and ASIK (for non-communicable disease services and immunization), which were not interoperable. Additionally, they must also manually record the data.

*“In cluster 2, we first have the SIGIZI [and] E-Kohort [an application for maternal and child health services]. As for manual registers, we have the infant and toddler cohort, which we combine to compile that data. Afterwards, we also gather data on women of reproductive age from there. We also collect data from Posyandu.”*

*(Health worker, Non-pilot Puskesmas 05, May 2025)*

The reporting mechanism still relied on a monthly tiered manual system. *Puskesmas* compiled data reported by UPKDK health workers and submitted it to the DHO. The reporting process generally used online instant messaging platforms, such as WhatsApp, and files were attached in a spreadsheet format.

*“Every month, the village midwife reports to us. We also report to the health center every month via WhatsApp and also send an Excel file.”*

*(Health worker, Non-pilot Puskesmas 05, February 2024)*

### Governance, leadership, and management

The lack of support from leaders and key stakeholders outside the health sector was a recurring theme throughout the study. At the district level, ILP was considered the sole responsibility of the DHO. At the village level, leaders were often unaware of their crucial role in promoting ILP and the health sector. Participants attributed this situation to the absence of strategic guidance and decisions from senior leadership. Participants expected the regents and the district secretariat to assume the leadership role and facilitate the involvement of stakeholders outside the health sector to support ILP implementation.

*“I strongly agree that ILP is part of the [PHC] transformation. All leaders should be involved and contribute. For a program to be considered successful, all levels [of stakeholders] must be involved. The leaders should be able to accommodate the sectoral units under them. So, they need to have the power to do so. If we, the DHO, invite other district agencies [to a meeting], since we are at the same level, they may not necessarily attend. If they do, they may send their staff [instead of the decision-makers]  ... even [sometimes] staff at the lowest level. This is because we do not hold the same power as the regent, or at least the District Secretary, who is the ‘principal’ of civil servants.”*

*(DHO staff, May 2025)*

Besides the lack of multisectoral support, district political dynamics created challenges in the governance of ILP. The change in district leadership, for instance, led to staff turnover at both the DHO and facility levels. Some staff members, who were rotated to other facilities or institutions, played key roles in establishing ILP in the district. However, their skills and knowledge regarding ILP were rarely documented and institutionalized. This caused a loss of learning and knowledge transfer and set back the development of ILP.

*“It is true, we formed a coordinating team to implement the ILP in West Sumbawa, signed by the regent and chaired by the regional secretary, in 2024. However, due to the changes in the regional head and deputy regional head in 2025, the team will be disbanded.”*

*(DHO staff, May 2025)*

Inconsistencies in supporting and understanding ILP were observed not only among non-health sectors and village leaders but also within the DHO. Both DHO and *Puskesmas* participants acknowledged that only a designated team for ILP within the DHO understood the essence of service integration. However, other DHO divisions were not well-versed in ILP and still thought and communicated using a program-based approach, without fully understanding that ILP was intended to reduce programmatic silos.

*“The human resources department [of DHO] has started using a cluster system and so has the public health division. But the disease control and environmental health division still frequently uses the term program, Sir. And all of this ultimately hampers ILP. This cluster [system], Sir. Where is the position [for example] of the person responsible for non-communicable disease control? Where does their role fit within this cluster structure? [Eventually], I always have to inform my colleagues that [in the ILP model], the diseases are categorized based on age-specific clusters.”*

*(Health worker, Non-pilot Puskesmas 06, May 2025)*

Challenges in managing ILP were also experienced by *Puskesmas* as the coordinator of primary care providers within their respective catchment areas. Under the ILP organizational structure, *Puskesmas* now has a division designated for management. This division, called the management cluster, serves as the administrative foundation of *Puskesmas* and coordinates functions such as human resources, financing, supplies, information systems, network coordination, community empowerment, and quality assurance [10]. Many *Puskesmas* workers assigned to this cluster, however, were not familiar with administrative and management matters. They admitted difficulties in performing their tasks since they lacked formal management backgrounds.

*“From this perspective of, what is it called? The management cluster, which includes six coordinator positions, covers quality assurance, provider] network management, and information systems. Those assigned to this cluster still need to learn everything from scratch. I understand that this cluster is somewhat difficult [to implement].”*

*(Health worker, Non-pilot Puskesmas 06, September 2024)*

### Regulation and guidelines

Our study identified inconsistencies in central government regulations. In September 2024, participants observed a significant discrepancy concerning the *Puskesmas* organizational structure as mandated by the ILP guideline (issued through the Minister of Health Decree Number 2015/2023) and the Minister of Health Regulation Number 43/2019 on *Puskesmas*. In implementing ILP, *Puskesmas* were required to change their organizational structure into five functional clusters (management, maternal and child health services, adult and elderly services, communicable disease control, and cross-cluster). However, during data collection in September 2024, the *Puskesmas* accreditation procedure still used the old organizational structure, which was based on two service categories: community and individual health services.

*“Yes, so we exchanged ideas with a Puskesmas [accreditation] surveyor about what they looked for [in an accreditation process], while we consulted with other Puskesmas here, including the pilot Puskesmas. We listened together to what they said. ‘Yes, it’s okay,’ that is what they [another Puskesmas] said, ‘It’s okay, sir, to use the new structure, use the ILP one.’ But, in the end, this [accreditation] surveyor, including our dentist who is also an accreditation surveyor, still suggested using the old structure, which follows The Minister of Health Regulation Number 43. So, for example, if the assessment element requires a position called “community health coordinator” [which should no longer exist in the ILP organizational structure], the surveyors must comply with it. So, in the end, we still used the old structure.”*

*(Health worker, Non-pilot Puskesmas 06, September 2024)*

Another gap was the absence of cross-ministerial regulation concerning UPKDK. ILP consolidates all village-level facilities into a single entity called UPKDK, which functions as the support unit for *Puskesmas*. This created a funding issue because village-level facilities were previously financed either by the DHO through *Puskesmas* or by village leaders. The creation of an integrated UPKDK raised the question of who should fund it. Participants expected the MOH, Ministry of Home Affairs, and Ministry of Village Affairs to issue a cross-ministerial regulation to clarify this matter.

*“A joint decree is needed, not only from the MOH, but also from the Ministry of Home Affairs and the Ministry of Villages, with the collective goal of service integration. So, with the central government’s regulations supporting this program, we can coordinate more seamlessly at the sub-district and village levels, because they [the sub-district and village leaders] also have their own regulatory umbrellas to support this program.”*

*(DHO staff, September 2024)*

The implementation of ILP in WSD was also hampered by an operational disconnect from the national technical guideline issued by the MOH. Participants found the guideline too lengthy and overloaded with information, making it difficult to comprehend. Although the document contained a lot of information, it was considered unclear and lacked actionable guidance. Consequently, some participants viewed ILP as merely administrative rather than transformative because the guideline did not clearly define what was meant by “integration” in ILP. This issue was compounded by the absence of a district-level guideline that would adapt and contextualize the national guideline into local practices.

*“The four functions (ILP) are still limited to administrative requirements. There is a feeling of being forced before they are fully developed, which impacts both quality and quantity. Technical guidelines from the Minister of Health Decree Number 2015 of 2023 exist, but the details and follow-up to those guidelines have yet to be implemented. For example, capacity building, human resource provision, infrastructure provision, involvement, support, and commitment from local and cross-sector governments have not been implemented as we would like.”*

*(DHO staff, September 2024)*

Gaps in local translation of national policies were also evident in the implementation of screening initiatives. By the final round of data collection (May 2025), there were no accounts regarding district regulations or guidelines that formally harmonized the ILP screening packages with the national screening initiative. Health workers also raised concerns regarding activity duplication and the lack of coordination between the two initiatives.

*“There are a lot of components [in the new screening initiative]. The program also comes with a new application [to record screening data]. It is unfortunate that they introduce a new screening program, whereas the current [ILP] one has not been implemented well. I find it confusing how these two initiatives can be synchronized.”*

*(Health worker, Non-pilot UPKDK 05, May 2025)*

### Financing

Financial inflexibility was seen as a major obstacle for *Puskesmas* to implement ILP. Despite holding a financial autonomy status to manage their funds and generate revenue, *Puskesmas* in WSD still lacked financing flexibility. Since the regent of WSD imposed a free health care regulation for all locals, *Puskesmas* could not generate revenue from retribution. As a result, *Puskesmas* relied heavily on fund transfers from the national health insurance scheme (called *Jaminan Kesehatan Nasional*) and the central government, mainly in the form of health operational assistance (*Bantuan Operasional Kesehatan/*BOK). *Jaminan Kesehatan Nasional* was focused on curative services, while BOK was allocated for promotive-preventive services with certain restrictions. Because of this fragmentation, *Puskesmas* faced difficulties in balancing promotive-preventive and curative services. Moreover, BOK menus remained organized around health programs, which contradicts ILP’s goal to reduce program siloes.

*“So, if I encounter an issue at the integrated health post [Posyandu] and realize, ‘I need to address stunting today,’ I am unable to reallocate funds from other budget lines because the budget nomenclature is already fixed.”*

*(Health worker, Non-pilot Puskesmas 06, May 2025)*

On top of that, UPKDK and *Posyandu* continued to be under-resourced. UPKDK, in particular, lacked the autonomy to manage its own budget, relying instead on the village and *Puskesmas* budgets. This dependence on village funding also impacted UPKDK’s infrastructure and human resource availability. Despite the district regulation mandating the allocation of village funds for health affairs, village leaders showed varying levels of political will to finance UPKD. As a result, some UPKDK still faced resource shortages.

*“[For UPKDK equipment] We were told to seek help from the Puskesmas. [On the other hand], the village facility belongs to the village, so it is not appropriate for us to ask the Puskesmas for the equipment. There are some things we can ask for, but they cannot all be procured, for example, sterilization equipment. We can budget for it through the village. But that is for health affairs, so it was really difficult to ask the village [government] back then. Not sure how it goes now.”*

*(Health worker, Non-pilot UPKDK 04, September 2024)*

### Physical resources

Participants reported challenges in pharmaceutical management at the UPKDK level. Since UPKDK did not have the authority to procure, they heavily depended on *Puskesmas* to procure, record, and report their pharmaceutical supplies. When UPKDK ran out of stock, the coordinators must submit a request to their respective *Puskesmas*. However, the quantity of drugs supplied to the UPKDK was limited.

*“Patients who are not covered in the National Health Insurance’s Chronic Disease Control Program are still allowed to get their medicines at the village clinic. However, the amount provided is limited. For instance, here at Puskesmas we can give them medicines for 10 days, but at the village clinic, they will only get supplies for 5 days. So, the next time they come to the village clinic, if their blood pressure remains high, they will be referred here, to the Puskesmas, for further treatment.”*

*(Health worker, Pilot Puskesmas, September 2024)*

Another issue related to pharmaceutical management in UPKDK is the lack of clear guidance. During the midline evaluation, participants highlighted the vague requirement about pharmaceutical storage and dispensing in UPKDK. Some UPKDK also stored and dispensed amoxicillin without a prescription.

*“In terms of pharmaceuticals, we are still confused about which medicines can be stored [at UPKDK] because there are no restrictions or regulations until now… They said it should depend on the internal policies [of Puskesmas] because there is no clear guidance about this. So, currently, we decide to base it on internal agreement only.”*

*(Health worker, Non-pilot Puskesmas 02, September 2024)*

UPKDK experienced issues in the availability and use of medical equipment and consumables. For example, all UPKDK involved in this study reported shortages of cholesterol test strips. Furthermore, although the DHO provided UPKDK with new anthropometric kits and blood pressure monitors, some health workers in UPKDK admitted they were not familiar with them.

*“We measure weight using the digital scale provided by the DHO. Because according to the ILP policy, we are required to use digital measurement only, although, we think, digital devices are not always accurate. Take the blood pressure monitor, for instance. If we use a manual one, this person does not have hypertension. But when we use a small, digital one, their blood pressure suddenly rises, from the usual 80-90 mmHg to 140 mmHg. So, sometimes we reduce the results from digital measurement by 20 points.”*

*(Health worker, Non-pilot UPKDK 01, March 2024)*

Infrastructure issues were observed across all three levels of primary care providers. During our data collection in March and September 2024, *Puskesmas* mentioned experiencing occasional power outages. Since they did not have an uninterruptible power supply, the quality of temperature-sensitive materials, such as samples for clinical examination, drugs, and vaccines, was compromised.

*“The problem is, we have a blood sample taken from the patient, but we cannot test it [because of a power outage], or we are about to do it, but the power goes out, and the sample is damaged, [whereas] the patient has been waiting.”*

*(Health worker, Pilot Puskesmas, March 2024)*

The issues were more pronounced in UPKDK and *Posyandu* because these facilities heavily relied on village budgets. Some UPKDK and *Posyandu* reported lacking proper water, sanitation, and hygiene facilities. While UPKDK was supplied with medical equipment and pharmaceuticals by *Puskesmas* and DHO, all UPKDK participating in this study did not have referral transport vehicles. Instead, they relied on cars provided by the village government or residents. Additionally, some UPKDK clinics were not equipped with motorcycles for home visits and outreach activities. Instead, the clinics relied on private vehicles owned by health workers or CHWs. Regarding the *Posyandu*, some reported not having designated rooms or buildings for patients to wait in line comfortably.

*“The Posyandu toilet is not functioning due to a lack of water. The water source is available, but there is no borewell. The [pumping] machine is also missing.”*

*(Posyandu CHW, Non-pilot UPKDK 01, March 2024)*

### Perceived facilitators

#### Local government commitment.

Support from local government at both district and village levels was a key factor in implementing ILP in WSD. During the pilot phase in 2022, the district government issued a decree instructing all district agencies to support ILP, along with a district regulation serving as a legal framework for UPKDK.

*“To date, we have issued four regulations supporting ILP activities. One is Regulation Number 1677 concerning the establishment of Posyandu Prima [the former name of UPKDK]. Previously, Posyandu Prima existed before the issuance of Minister of Health Decree Number 15 of 2023. We then issued a Regent’s Instruction to all relevant district government agencies, including the Regional Development Planning Agency [Bappeda], to support the ILP in West Sumbawa, both through financing and other forms of support.”*

*(DHO staff, March 2024)*

The district and village governments also provided financial support, mainly from transfer funds from the national governments, to expand ILP to non-pilot *Puskesmas* by equipping UPKDK with new equipment and hiring human resources, namely nurses, midwives, and CHWs, to be placed at UPKDK.

*“Village clinics are under the authority of village governments. However, they were built through the contribution of the district government. The human resources of village clinics can be financed through the village budget and through the regional budget because on average, health workers at this health center are civil servants or contract workers who are funded by the district budget.”*

*(DVCEO staff, September 2024)*

Since UPKDK is considered a significant innovation in ILP, participants regarded the support from village heads as crucial for implementing ILP.

*“[The key drivers are] the village leader as well as strong cross-sectoral collaboration. As much as 40% of the village funds are for health. ‘What else does the village clinic want?’ they [the village government] asked us. ‘What other innovations do you want? What do you want to do?’”*

*(Health worker, Pilot UPKDK, March 2024)*

#### Support from community health workers.

Despite various challenges in providing incentives for *Posyandu* CHWs from the village government, CHWs continued to participate in implementing ILP. *Posyandu* CHWs did not only work during the operational hours of health posts; instead, they played key roles in supporting LAM, as they were regarded as the “eyes on the ground” by *Puskesmas* and UPKDK.

*“As for me, I also participate [in LAM], Ma’am. I often participate. Usually, [these activities] are centered at the village clinics, and when I go to the houses, I am usually accompanied by a CHW. So, the CHW also acts as an ‘eye on the ground’ [for the Puskesmas] in the village, Ma’am. For example, if a community member shows tuberculosis symptoms, we immediately ask the CHW, ‘This is the patient [with the symptoms]. Can you check them out?’ Something like that.”*

*(Health worker, Non-pilot Puskesmas 02, September 2024)*

In working with the communities, *Posyandu* CHWs collaborated with *Agen Gotong Royong* (AGR), community empowerment cadres employed by the district government. AGRs bridged communication between the district government and the communities. AGR also collected community data, which could support *Posyandu*, UPKDK, and *Puskesmas* to establish their surveillance database.

*“But with the presence of AGRs, I see that our data is getting stronger. For instance, the population size in our catchment area is 25, no, almost 26 [thousand]. When I asked about the community database collected by the AGRs, it contained approximately 23 [thousand]. I think the data covers almost all of our target population. I can use it to determine our target number [for services].”*

*(Health worker, Non-pilot Puskesmas 06, May 2025)*

#### Support from the Ministry of Health.

Participants regarded hands-on supervision and capacity building from the MOH, who developed the concept and guidelines for ILP, as particularly crucial for its implementation. During the piloting phase of ILP, MOH assigned several representatives to WSD to offer technical guidance to *Puskesmas* and UPKDK.

*“In 2023, almost every month [the MOH visited us]. At least 1-2 representatives were sent to provide technical assistance.”*

*(Health worker, Pilot UPKDK, March 2024)*

Technical support from MOH, however, was concentrated primarily on pilot *Puskesmas* and UPKDK. The same level of support was not experienced by non-pilot facilities.

*“In my opinion, the implementation [of ILP] at the pilot Puskesmas was good because they were directly guided by the MOH for several months. They were guided until their implementation was considered successful. In our case, we did not receive the same guidance.”*

*(Health worker, Non-pilot Puskesmas 02, September 2024)*

## Discussion

This study examined the implementation of IPHC through the ILP approach in Indonesia, a country with a complex and decentralized health system. The study was conducted in a district designated as a national pilot ILP site. We used a qualitative, thematic analysis design and employed a realist paradigm, whereby meaning was grounded on participants’ accounts [26]. Our understanding of service integration was guided by two complementary models. Shortell et al. conceptualize integration as physician-system, functional, and clinical, with clinical integration considered the most pivotal for healthcare system performance [6]. Singer et al. expands this framework by identifying structural, functional, normative, interpersonal, and process dimensions of integration [7]. Together, these models inform that a successful service integration requires the alignment of human resources, organizational infrastructures, and culture, alongside effective collaboration and coordination. In addition, we adapted the MRC/NIHR Framework for Complex Intervention Evaluation [41] to define key elements of intervention implementation and to guide thematic development. Our findings highlight the importance of structural and operational redesign, as well as a deep understanding of local social, political, and organizational contexts for establishing IPHC in a decentralized system.

Our examination of district participants’ views of the acceptability and feasibility of ILP as an IPHC model yields a generally positive remark. Participants expressed familiarity with ILP’s life-cycle approach since it was seen as similar to an existing local program (*Gotong Royong*), which aimed to offer accessible and responsive public services through community integrated posts for people of all ages. Participants noted that ILP brought services closer to the community via village and community-level facilities (UPKDK and *Posyandu*, respectively). However, slower adoption of ILP was observed in non-pilot facilities, where participants cited the lack of practical supervision and the lengthy, information-dense national guidelines as reasons for this. Therefore, challenges in non-pilot facilities do not reflect participants’ resistance to ILP but are primarily attributed to technical and operational setbacks. Nonetheless, by the end of data collection, all *Puskesmas* already implemented ILP, although to different extents. Additionally, some participants viewed the current ILP implementation as primarily administrative rather than transformative, since program-based implementation persisted. Increased workload at village and community levels due to expanded screenings and higher health care demand was also emphasized by some pilot and non-pilot facility participants. Our findings align with other studies in Indonesia. A study on the readiness of *Posyandu* for ILP in West Java, Indonesia, involving DHO staff, health workers, and CHWs, showed similar results, with most participants perceiving ILP as a suitable method to deliver services across all age groups and to connect primary care providers [42]. Like our findings, the study also highlighted concerns about ILP implementation, such as increased workload for *Posyandu* CHWs [42].

Our analysis demonstrated that leadership and commitment at the local level, as well as multi-sectoral governance, played key roles in district-level IPHC implementation within a decentralized context. Since the structure of PHC in Indonesia relies on support from health and non-health sectors [43], the lack of political commitment from key actors outside the health sector at the central and sub-national levels, undermined ILP implementation at the district level. This finding echoed the insights from IPHC implementation in Nigeria which stressed the importance of continuing advocacy to sub-national governments, who hold the authority of implementation, for successful implementation of national policies of IPHC at the local level [44]. The study also suggested that recognizing and engaging with the supreme decision-makers that had the authority over other sectors were pivotal for the process [44].

Our results also showed that financing issues in the form of PHC financing source fragmentation and a lack of a pooling mechanism in the study district further complicated local IPHC implementation. On top of that, the issuance of district free health care policy hindered *Puskesmas* from increasing their revenue to equitably fund promotive-preventive and curative services, despite having the flexible financial autonomy status. Drawing on the integration model by Singer et al. [7], these findings provided an example of issues in the functional aspect of integration. They also showed that sub-national PHC financing in Indonesia is shaped by complex political dynamics of stakeholders in the system. According to Gatome-Munyua et al., defragmenting financing in a complex PHC system should be treated as a long-term, iterative, system-wide initiative and, as such, political will is crucial for its effectiveness [45].

Our paper demonstrated that challenges in health system functions, including human resources, infrastructure, supplies, and information systems, hindered a full implementation of IPHC at the district level. In WSD, the operational levers of PHC (human resources, data management, and physical resources) were not sufficient to support ILP implementation. Both pilot and non-pilot facilities faced scarcity of medical personnel and CHWs, as well as inadequacy of human resource capacity, resulting in the failure to deliver comprehensive and coordinated services. This was largely driven by the lack of comprehensive hands-on capacity building, which was more pronounced in non-pilot facilities.

Notably, gaps in human resource competence were observed in pilot and non-pilot UPKDK clinics, particularly regarding antibiotic dispensing practices. The persistence of this issue in the pilot facility despite their earlier exposure to ILP training, supervision, and implementation support indicate that capacity-building alone is not sufficient to address this challenge. The dispensing of antibiotics without a physician’s prescription was reported across pilot and non-pilot UPKDKs, largely attributed to unclear guidelines about pharmaceutical storage and dispensing at village-level facilities. This practice raises patient safety concerns, as inappropriate antibiotic consumption leads to the development of antimicrobial resistance (AMR) [46]. Although community pharmacies have been identified as the primary site of non-prescription antibiotic dispensing in Indonesia [47,48], our finding suggests that UPKDK clinics may emerge as a novel source of inappropriate antibiotic distribution if this operational ambiguity persists.

We also identified an issue with the introduction of medical technologies at the UPKDK level. A participant from a non-pilot UPKDK clinic reported difficulty in using a newly introduced digital blood pressure measurement tool supplied by the DHO. The device was perceived to consistently produce higher readings than the old one. As such, blood pressure values were sometimes adjusted downward by 20 mmHg based on the health worker’s judgment. This example highlights insufficiency in training, standardized measurement protocols, device validation mechanisms, and technical support for rural health workers when new technology is introduced. Such gaps may compromise not only treatment accuracy, but also data quality in the information system, which is essential to support structural integration [7].

The variable, yet fragmented and not interoperable digital applications for data management and reporting, as reported by frontline health worker participants, further created complexity in establishing an integrated information system. Singer et al. emphasize that the lack of interoperable information systems may undermine process integration as it hinders information sharing [7]. Our results are aligned with previous research in Indonesia, which identified CHW shortage and educational disparity, limited infrastructures, and scattered digital reporting as the primary barriers for *Posyandu* to implement ILP [42].

Challenges related to physical resources, pharmaceutical supply and equipment, as well as water and electricity were identified at all primary care provider levels regardless of the pilot status. The persistence of these constraints in pilot and non-pilot facilities further indicates that barriers in ILP scale-up are more closely related to discrepancies in learning and capacity-building support than with resource availability. Overall, our results suggested that establishing IPHC requires coordinating public health functions, which is aligned with the principles of an effective model of care introduced by the World Health Organization and the United Nations Children’s Fund [3].

This study identified the support of local government, CHWs, and MOH as vital enablers for implementing ILP. Our findings align with the recommendations from Amon et al. in Ghana, which emphasize the importance of engaging national and local health structures to strengthen resource distribution and utilization [49]. However, it is worth noting that the capacity of decision-makers is influenced by decision spaces and autonomy [50]. In terms of CHW roles, our study has once again shown their essential contributions in strengthening IPHC, as highlighted by Shrestha et al. [51]. CHWs helped expand PHC coverage and improved community health [51]. In the Indonesian context, CHWs are acknowledged as key resources for community engagement, health education, and delivering integrated services through *Posyandu* [43], which serve as the backbone of PHC at the community level.

Our findings suggested the importance of redefining “integration” within the IPHC concept. Drawing from the lessons of WSD, the implementation of IPHC in Indonesia through the ILP approach was still heavily focused on the administrative and input aspects, with few emphases on the social and process aspect of integration. Shortell et al. argued that effective integration requires not only operational levers, such as financing and information system, but also personnel that share organizational missions and values, as well as strategic planning and continuous improvement processes across the entire system [6]. Singer et al. further suggested that interpersonal integration among those involved in patient care, including the patients themselves, is pivotal to achieving care outcomes, such as efficiency and satisfaction [7]. In summary, integration requires coordinated human resources, functions, activities, and operating units [6,7] and, thus, should not be viewed as the end state, but rather as a process to achieve outcomes. These outcomes may include improved care coordination, continuity, quality, efficiency, accountability, and better patient experiences [7]. This understanding needs to be further translated into a clear theory of change encompassing the structure, input, process, output, and outcomes to facilitate various stakeholders understand, implement, and measure ILP.

### Strengths and Limitations

Our study has several limitations. First, the findings are not generalizable to other regions of Indonesia since it was conducted in a pilot district. However, the study generated rich thematic qualitative findings which can be contextualized in other settings. Our study also included non-pilot facilities to provide more diverse insights on the rollout of IPHC. Second, some FGDs and interviews in November 2023 were conducted online. While online data collection limits non-verbal interaction with participants [52], the methods were the most accessible due to time and resource constraints during the period. However, our subsequent data collection activities were conducted face-to-face, significantly improving the richness of findings from online data collection. The repeated data collection also helped us build rapport with our participants. Third, the study is conducted in Indonesia and focused on the health system factors shaping ILP implementation at the district level rather than the effect of ILP on patient, service, and population outcomes. Consequently, while outcomes such as continuity of care, coordination, comprehensiveness of care, accountability, or service delivery stewardship are considered important aspects of IPHC, they were beyond the scope of this paper. However, insights on how system-wide factors shape the implementation of IPHC at the-subnational level have broader implications on PHC performance and can be transferable to decentralized countries with similar contexts.

### Recommendations for policy, practice, and future research

By portraying IPHC as a systemic reform, our study highlighted several implications pertaining to the decentralization context. First, the central government’s integration model needs contextualization at the subnational level. The absence of local guidelines for ILP and regulations harmonizing ILP screening packages with the new national screening program suggests that future policies should provide subnational governments with clear operational pathways for integrating overlapping programs and initiatives. The subnational implementers should also be provided with flexibility to tailor the model in accordance with local needs, strengths, and challenges [53]. The learnings from subnational implementation should also serve as feedback for the national government to continuously fine-tuning the ILP guidelines and model. Second, integration between care providers would not be realized if leaders across various levels and sectors remain siloed [50]. The support of leaders outside the health sector is also crucial. Our study found fragmentation in reporting and information systems attributable to the lack of collaborative governance, resulting in sub-optimal data quality and utilization for decision-making. A clear chain of command at the national, provincial, district, and cross-level governments is necessary to prevent fragmentation in the governance [43]. Fourth, effective scale-up of IPHC requires broader health system strengthening [54,55]. The reform should simultaneously address strategic and operational building blocks to create an enabling system for governments, providers, and societies for establishing equitable, comprehensive, and integrated health services [3]. Finally, IPHC should not be perceived as an end in itself, but as a mechanism to strengthen PHC functions to achieve desired outcomes. Therefore, future policies should have clear, formalized visions and theory of change as frameworks to guide initiatives in PHC strengthening [3].

Our study also highlights several priorities for future research. We recommend examinations of how IPHC contributes to better service integration, health system performance, and population outcomes. We also suggest exploring community perspectives on the implementation of IPHC, particularly about whether and how the new approach improves service accessibility. Finally, given the limited geographical scope of our study, future research should examine IPHC implementation across diverse spatial and socioeconomic settings.

## Conclusions

Based on Indonesia’s ILP experience in WSD, the main key takeaway from this study is that IPHC in decentralized systems requires more than conceptual acceptability. IPHC demands coordinated governance, regulatory coherence, and aligned financing across government levels and sectors. IPHC also requires interoperable information systems and adequate resources to ensure service comprehensiveness and continuity. Our study highlighted persistent fragmentation in the information systems, financing, regulatory frameworks, and vertical programs, which suggested that comprehensive PHC reform could not occur through health sector policies alone. Local government commitment and CHW engagement in the study district proved essential, but insufficient without national-level coordination to address systemic barriers. Finally, successful integration requires recognizing interdependencies among health system building blocks. For instance, service delivery integration depends on financing integration, which is shaped by governance coordination and regulatory alignment. Therefore, reforming PHC requires simultaneous multi-level reforms. These lessons extend beyond ILP in Indonesia to broader PHC strengthening efforts, which demonstrate common patterns of fragmentation and coordination failures across various aspects of decentralized health systems.

## Supporting information

S1 File Completed Consolidated Criteria for Reporting Qualitative Research (COREQ) Checklist. (DOCX)

S2 File Data collection topic guide. (PDF)

S3 File Summary of Themes and Subthemes. (PDF)
