# Supplementary material for: Barriers and enablers in implementing integrated primary health care: A qualitative assessment in West Sumbawa District, Indonesia
Source: PLOS Glob Public Health. 2026 Sep 11;6(9):e0007238. doi: 10.1371/journal.pgph.0007238 (PMC13567773; doi:10.1371/journal.pgph.0007238)
Supplement: Supplementary file 1 [file pgph.0007238.s002.pdf]

S2 File. Data collection topic guide

<u>Data Collection Phase 1 (November 2023)</u>

Date :

Time :

Location :

Participants :

Interviewer/Facilitator:

Note-taker :

Topic: Health system in West Sumbawa District

Questions	Response
Describe the characteristics of West Sumbawa District?	
Please explain the structure of the village clinics in West Sumbawa District.	
Describe the implementation of *posyandu gotong royong* and family-based integrated health service posts in villages (*posyandu keluarga*) in West Sumbawa District.	
Please tell us about the health worker in West Sumbawa District.<br>a. What types of health workers remain insufficient in West Sumbawa Regency?<br>b. Are there any types of health workers that exceed the required number?	
Describe the implementation of cluster-based services under the ILP approach at *Puskesmas* in West Sumbawa District.<br>a. What were the challenges in the implementation?<br>b. If there were challenges, how were they addressed?	

<u>Data Collection Phase 2 (February 2024)</u>

Date :

Time :

Location :

Participants :

Interviewer/Facilitator:

Note-taker :

Topic: Health Service Performance at *Puskesmas* in West Sumbawa District

Questions	Response
Describe the health service delivery system at this *Puskesmas*.<br>a. Please describe the characteristics of the population served by this *Puskesmas*<br>b. Please describe the primary care provider network of this *Puskesmas*, including the number of village clinics (owned by *Puskesmas* and village governments), as well as *Posyandu*<br>c. Please describe the health workforce situation at this *Puskesmas*	
Please describe maternal and child health services at this *Puskesmas*.<br>a. What are the challenges in implementing maternal and child health services?<br>b. How are data collection and reporting processes for maternal and child health services carried out?<br>c. In the implementation of ILP, what are the key differences with regards to maternal and child health services before and after the integration?	
Please describe health services for productive age and elderly people at this *Puskesmas*.<br>a. What initiatives have been taken to increase the coverage of screening services for productive age and elderly people?<br>b. How are screening services for productive age and elderly people being reported?<br>c. What are the enabling factors and challenges in the implementation of these services?<br>d. Have health services for productive age and elderly changed before and after the implementation of ILP?	

Please describe communicable disease services at this *Puskesmas*.<br>a. What are the most common communicable diseases at this *Puskesmas*?<br>b. What are the enabling factors and challenges in the implementation of communicable disease screening services?<br>c. Have communicable disease services changed before and after the implementation of ILP?	
Please describe cross-cluster services at this *Puskesmas*.<br>a. Are there any challenges related to the availability of essential medicines?<br>b. How does the primary health care center ensure the availability of medicines?<br>c. Are the laboratory facilities at this *Puskesmas* adequate?<br>d. Are there any specific challenges related to laboratory services?<br>e. If there are challenges related to laboratory services, how were these challenges addressed?	

<u>Data Collection Phase 3 (March 2024)</u>

Date :

Time :

Location :

Participants :

Interviewer/Facilitator:

Note-taker :

Topic: Initial Implementation of ILP in West Sumbawa District

Questions	Response
Please tell us about the current state of ILP implementation in this district.	
Please tell us, in chronological order, about the evolution of ILP implementation in this district, starting from the piloting phase.	
To what extent have you been participating in ILP in this district?	
In your observation, what components of health system in this district **remain** after the launch of ILP?	
In your observation, what components of health system in this district **change** after the launch of ILP?	
What do you think are the enabling factors of ILP implementation in this district?<br>(*Note to facilitator: please use the following factors to guide the discussion*<br>*a. Technical guidance from the Ministry of Health*<br>*b. Technical guidance developed by the district government (if available)*<br>*c. Human resources*<br>*d. Financing*<br>*e. Physical resources*<br>*f. Geographical access*<br>*g. Pharmaceutical and vaccine supplies*<br>*h. Medical equipment supplies*<br>*i. Information systems*<br>*j. Referral transport*<br>*k. Referral communication*<br>*l. Commitment from district leaders*<br>*m. Commitment from village leaders*<br>*n. Commitment from community health workers*<br>o. *Community participation*)	

What do you think are the barriers of ILP implementation in this district?<br>(*Note to facilitator: please use the following factors to guide the discussion*<br>*a. Technical guidance from the Ministry of Health*<br>*b. Technical guidance developed by the district government (if available)*<br>*c. Human resources*<br>*d. Financing*<br>*e. Physical resources*<br>*f. Geographical access*<br>*g. Pharmaceutical and vaccine supplies*<br>*h. Medical equipment supplies*<br>*i. Information systems*<br>*j. Referral transport*<br>*k. Referral communication*<br>*l. Commitment from district leaders*<br>*m. Commitment from village leaders*<br>*n. Commitment from community health workers*<br>*Community participation*)	
For each barrier mentioned, what mitigation strategies have been in place to overcome it?	
What do you think about the technical guidance developed by the Ministry of Health? (in terms of clarity, actionability, and feasibility to translate into district-level guidance)	
Is there a district-level guidance for ILP in this district?<br>a. If there is, who participated in the process?<br>b. How has it been communicated to healthcare facilities?<br>c. To what extent do healthcare facility staff understand the guidance?<br>d. What initiatives have been taken to ensure healthcare facility staff understand and implement the guidance?	
Is there a facility-level standard operating procedures for ILP in this district?<br>a. If there is, how was it developed?<br>b. Who participated in its development?<br>c. How has it been communicated to healthcare facility staff?<br>d. To what extent do healthcare facility staff understand the guidance?<br>e. What initiatives have been taken to ensure healthcare facility staff understand and implement the guidance?	

With regards to information systems:<br>a. Has there been any training or supervision to strengthen information systems for ILP?<br>b. How has the training or supervision helped in the development of information systems in this district/facility?<br>c. What do you think is needed to ensure that information systems can support integration?	
In your observation, what components of ILP have been appropriately implemented in this district/facility?	
In your observation, what components of ILP have **not** been appropriately implemented in this district/facility?	
In your observation, have there been any positive outputs or outcomes resulted from ILP?<br>(*Note to facilitator: please use the following list of potential outputs and outcomes to guide the discussion*<br>a. *Indicator achievement*<br>*b. Level of commitment from key stakeholders*<br>*c. Number of facilities adopting ILP)*	
For each result mentioned, what factors do you think contribute to the achievement?	
With regards to the scale-up of ILP, how likely would non-pilot *Puskesmas,* UPKDK, *Posyandu,* and villages accept and adopt ILP in the future?	
Could you tell us your plans for the next six months, with regards to the following aspects?<br>a. Ensuring the compliance of ILP implementation to the guidance from the Ministry of Health<br>b. Ensuring that ILP can be implemented across all provider levels<br>c. Ensuring that available resources can be optimized to support ILP<br>d. Ensuring the effectiveness of ILP<br>e. Scaling up ILP in non-pilot facilities<br>f. Other plans	

<u>Data Collection Phase 4 (September 2024)</u>

Date :

Time :

Location :

Participants :

Interviewer/Facilitator:

Note-taker :

Topic: Progress in ILP Implementation in West Sumbawa District

**Questions**	**Response**
Please tell us about the current state of ILP implementation in this district, including any changes observed since March 2024.	
Are there any components that have not changed since March 2024?	
What do you think are the enabling factors of ILP implementation in this district?<br>(*Note to facilitator: please use the following factors to guide the discussion*<br>*a. Regulatory support*<br>*b. Technical guidance from the Ministry of Health*<br>*c. Technical guidance developed by the district government (if available)*<br>*d. Human resources*<br>*e. Financing*<br>*f. Physical resources*<br>*g. Geographical access*<br>*h. Pharmaceutical and vaccine supplies*<br>*i. Medical equipment supplies*<br>*j. Information systems*<br>*k. Referral transport*<br>*l. Referral communication*<br>*m. Commitment from district leaders*<br>*n. Commitment from village leaders*<br>*o. Commitment from community health workers*<br>p. *Community participation*)	
What do you think are the barriers of ILP implementation in this district?<br>(*Note to facilitator: please use the following factors to guide the discussion*<br>*a. Regulatory support*	

b. Technical guidance from the Ministry of Health<br>*c. Technical guidance developed by the district government (if available)*<br>*d. Human resources*<br>*e. Financing*<br>*f. Physical resources*<br>*g. Geographical access*<br>*h. Pharmaceutical and vaccine supplies*<br>*i. Medical equipment supplies*<br>*j. Information systems*<br>*k. Referral transport*<br>*l. Referral communication*<br>*m. Commitment from district leaders*<br>*n. Commitment from village leaders*<br>*o. Commitment from community health workers*<br>*p. Community participation*)	
For each barrier mentioned, what mitigation strategies have been in place to overcome it?	
What do you think about the technical guidance developed by the Ministry of Health? (in terms of clarity, actionability, and feasibility to translate into district-level guidance)<br><br>*(Note: this question is asked to observe changes in perceptions regarding the technical guidance)*	
Is there a district-level guidance for ILP in this district?<br>a. If there is, who participated in the process?<br>b. How has it been communicated to healthcare facilities?<br>c. To what extent do healthcare facility staff understand the guidance?<br>d. What initiatives have been taken to ensure healthcare facility staff understand and implement the guidance?	
Is there a facility-level standard operating procedures for ILP in this district?<br>a. If there is, how was it developed?<br>b. Who participated in its development?<br>c. How has it been communicated to healthcare facility staff?<br>d. To what extent do healthcare facility staff understand the guidance?<br>e. What initiatives have been taken to ensure healthcare facility staff understand and implement the guidance?	

In your observation, what components of ILP have been appropriately implemented in this district/facility?	
In your observation, what components of ILP have **not** been appropriately implemented in this district/facility?	
In your observation, have there been any positive outputs or outcomes resulted from ILP?<br>(*Note to facilitator: please use the following list of potential outputs and outcomes to guide the discussion*<br>a. *Indicator achievement*<br>b. *Level of commitment from key stakeholders*<br>c. *Number of facilities adopting ILP)*	
For each result mentioned, what factors do you think contribute to the achievement?	
With regards to the scale-up of ILP, how likely would non-pilot *Puskesmas,* UPKDK, *Posyandu,* and villages accept and adopt ILP in the future?	
What do you think about the current ILP model implemented in your district? (In terms of relevance, feasibility, and refinements needed)	
Could you tell us your plans for the next six months, with regards to the following aspects?<br>a. Ensuring the compliance of ILP implementation to the guidance from the Ministry of Health<br>b. Ensuring that ILP can be implemented across all provider levels<br>c. Ensuring that available resources can be optimized to support ILP<br>d. Ensuring the effectiveness of ILP<br>e. Scaling up ILP in non-pilot facilities<br>f. Other plans	

<u>Data Collection Phase 5 (May 2025)</u>

Date :

Time :

Location :

Participants :

Interviewer/Facilitator:

Note-taker :

Topic:

a. Progress in ILP Implementation in West Sumbawa District

Questions	Response
Please tell us about the current state of ILP implementation in this district, including any changes observed since September 2024.	
Are there any components that have not changed since September 2024?	
Please describe service delivery models in this district that involve ILP primary care providers.<br><br>(*Note to facilitator: participants can use an A3 drawing paper, markers, and sticky note to describe the models)*	
What do you think are the enabling factors of ILP implementation in this district?<br>(*Note to facilitator: please use the following factors to guide the discussion*<br>*a. Regulatory support*<br>*b. Technical guidance from the Ministry of Health*<br>*c. Technical guidance developed by the district government (if available)*<br>*d. Human resources*<br>*e. Financing*<br>*f. Physical resources*<br>*g. Geographical access*<br>*h. Pharmaceutical and vaccine supplies*<br>*i. Medical equipment supplies*<br>*j. Information systems*<br>*k. Referral transport*<br>*l. Referral communication*<br>*m. Commitment from district leaders*	

n. Commitment from village leaders<br>*o. Commitment from community health workers*<br>p. *Community participation*)	
What do you think are the barriers of ILP implementation in this district?<br>(*Note to facilitator: please use the following factors to guide the discussion*<br>*a. Regulatory support*<br>*b. Technical guidance from the Ministry of Health*<br>*c. Technical guidance developed by the district government (if available)*<br>*d. Human resources*<br>*e. Financing*<br>*f. Physical resources*<br>*g. Geographical access*<br>*h. Pharmaceutical and vaccine supplies*<br>*i. Medical equipment supplies*<br>*j. Information systems*<br>*k. Referral transport*<br>*l. Referral communication*<br>*m. Commitment from district leaders*<br>*n. Commitment from village leaders*<br>*o. Commitment from community health workers*<br>*p. Community participation*)	
For each barrier mentioned, what mitigation strategies have been in place to overcome it?	
What do you think about the technical guidance developed by the Ministry of Health? (in terms of clarity, actionability, and feasibility to translate into district-level guidance)<br><br>*(Note: this question is asked to observe changes in perceptions regarding the technical guidance)*	
Are there any components from the Ministry of Health technical guidance that got adjusted in this district/facility?<br>a. If there are, what are those components?<br>b. Who participated in the process?<br>c. How has it been communicated to healthcare facility staff?<br>d. To what extent do healthcare facility staff understand the guidance?<br>e. What initiatives have been taken to ensure healthcare facility staff understand and implement the adjustment?	

In your observation, have there been any positive outputs or outcomes resulted from ILP?<br>(*Note to facilitator: please use the following list of potential outputs and outcomes to guide the discussion*<br>d. *Indicator achievement*<br>e. *Level of commitment from key stakeholders*<br>f. *Number of facilities adopting ILP)*	
For each result mentioned, what factors do you think contribute to the achievement?	
With regards to the scale-up of ILP, how likely would non-pilot *Puskesmas,* UPKDK, *Posyandu,* and villages accept and adopt ILP in the future?	
What do you think about the sustainability of ILP in this district?	
Could you tell us your plans for the next (a) six months and (b) one year, with regards to the following aspects?<br>a. Ensuring that ILP can be implemented across all provider levels<br>b. Ensuring that available resources can be optimized to support ILP<br>c. Ensuring the effectiveness of ILP<br>d. Scaling up ILP in non-pilot facilities<br>e. Sustaining ILP	

b. Health Service Performance at *Puskesmas* in West Sumbawa District

Questions	Response
**Health Service Performance at *Puskesmas* in West Sumbawa District**	
Please explain how each cluster at this *Puskesmas* collects routine data related to health service performance.	
Please explain the differences in routine data collection at this *Puskesmas* before and after ILP.	
How do village, subdistrict, and district governments provided support the implementation of ILP at this *Puskesmas*?	
