# Supplementary material for: Barriers and enablers in implementing integrated primary health care: A qualitative assessment in West Sumbawa District, Indonesia
Source: PLOS Glob Public Health. 2026 Sep 11;6(9):e0007238. doi: 10.1371/journal.pgph.0007238 (PMC13567773; doi:10.1371/journal.pgph.0007238)
Supplement: Supplementary file 2 [file pgph.0007238.s003.pdf]

S3 File. Summary of Themes and Subthemes

**Theme**	**Subtheme**	**Description**	**Excerpt**
Description of ILP Implementation	Familiarity with the ILP Concept	Similarity to a local approach that has long been running in West Sumbawa District, namely Gotong Royong *Posyandu*	"In terms of *Posyandu*, it is similar to our *Posyandu Gotong Royong* program. The new feature [of ILP], we think, is in the cluster-based system."
		UPKDK already provided services for all age groups before ILP but now is strengthened and made to be more comprehensive with the implementation of ILP.	"We have to manage everything, from health visits, sick visits, emergencies... all of them. From 0 month to 60 years old."
			"Our services are almost the same, Ma'am. The only difference is in the cluster system. She [the UPKDK midwife] used to be a midwife in the [*Puskesmas*] delivery room, so she already knew [how to handle the maternal and child health cluster]. Then, after another village midwife was transferred to the *Puskesmas*, she [the UPKDK midwife] was transferred here in her place. So, everything was already in place. When there was ILP, our job division was clear; ILP made it clearer."
	Adoption Process	Independent learning among non-pilot *Puskesmas* and UPKDK due to limited assistance and training related to ILP	"We did not receive mentoring based on clusters or units, which we think may facilitate better understanding. No. We were only invited in a discussion forum."
		ILP resulted in the shift from a program and service category-based model to a life cycle-based approach organized into five functional clusters. The facilities faced challenges in adapting to this change.	"After we are divided based on clusters, we still feel like we are responsible for certain programs. So, within each cluster, there are people who used to be program managers. These people still have trouble blending in their cluster. For instance, in cluster two, since we will be responsible for mothers, adolescents, and children, that means we have to master the services standards across all those targets. But some are still not used to the change from program-based [to the cluster system]."
		Some facilities struggled to manage longer queues and increased workload due to ILP's expanded screening	"Now it has gotten better. But, in the beginning, we had long queues, particularly when recording patient data. It was horrible, the staff was overwhelmed too."

Key Barriers	Human Resources	Scarcity of general physicians, dentists, medical technologists, and support staff, such as ambulance drivers	"According to the Minister of Health Regulation, we have a minimum of eight doctors at *Puskesmas*. But we only have three. So, those three are in cluster 2, 3, and inpatient care. We are still short [for doctors]."
		Difficulties in equipping UPKDK with at least two health practitioners and two CHWs	"So, I was once invited to a hearing at the district council, so I said to them, 'What should I do?' because I was having difficulties. Currently, with limited workforces, it is difficult to place staff in village facilities. My conclusion is that villages are not considered appealing by health workers."
		Challenges in recruiting and retaining *Posyandu* CHWs	"It's difficult [to recruit younger cadres] because we look for people who are suited for this position, who are willing [to work despite the little amount of incentive]."
		Health workers struggled to adapt to ILP's cluster-based system	"Our colleagues' mindsets… as midwives and nurses, they often think that their job is limited to providing clinical care; midwives who used to work with women who are pregnant, giving birth, post-pregnant, and babies are not familiar with other programs in *Puskesmas.* Now, all programs in *Puskesmas* have been transferred to the village clinics. Because of this, their roles in providing other services [based on the cluster] are not optimum yet. Sir, it is true there are still administrative mishaps here and there. It feels like we are just going around without reaching the goal. We must scale up, but we do not have enough supporting teams, only the core ones, whose skills are not ready to use yet."
		Limited digital literacy among *Posyandu* CHWs	"We already have five CHWs in each Posyandu. However, there have been many upgrades to the data entry application, and not all cadres are well-educated. You know, some are elementary school graduates [and therefore need to learn to use communication devices]."
	Information Systems	Increase in the number of data reporting applications required to be completed by health care workers	"We have ASIK, e-kohort, SIHA, and SIMKESWA for mental health. Each application is handled by their respective program manager, so the data is separated."
		No centralized system for collecting data used by *Puskesmas*	"For the reporting application, if possible, we suggest it should be submitted in one platform, so that there is no confusion, for example, like dengue fever, malaria, and we also have SKDR and so on, so it's all in one place, so it's like a cellphone, just press this menu and you

			want to report it, so we don't get confused. Now, there are lots of applications.”
		Manual data recording still needed	“So, there is still a backup if there is power outage, whether we like it or not, we not to draw the data manually first, such as by printing out the patient data.”
	Governance, Leadership, and Management	Limited support from leaders and key stakeholders outside the health sector	“Yes, as I said, when we tried to develop integration with people outside the health sector, they do not understand their role in ILP.”
		Lack of clear chain of command at the district level	“It turns out that a conductor is needed in the field to reduce the egoism of each manager, right? From what I understand, if there is a conductor, it means giving [the authority], because the ones who [actually] know the problems are the officers in the village, right? Meanwhile, since we only have at most one or two program managers in a *Puskesmas*, it is most likely that they do not know [everything in the field]. They are not in the field 24 hours a day.”
		Political dynamics affected ILP implementation	“It’s different… how am I supposed to say it... when the village head changes, the policy will change as well.”
		Inconsistency in supporting and understanding of ILP in DHO	“I spoke to the DHO about ILP, Sir. From the beginning, I have always criticized it. I only criticized simple things. The [invitation] letter [sent by the DHO] always used the old organizational structure before ILP, Sir, [such as] health promotion coordinator. We do not have a health promotion coordinator since our organizational structure follows ILP, not the old one. [When it happens] We are confused, who is the health promotion coordinator [in this *Puskesmas*]?”
		Lack of capacity in the management cluster	“When I looked at the ILP organizational structure, Ma’am, indeed some updates were required. For example, our administrative coordinator, after all, is from the DHO, but not a health professional. But when I looked at the guidelines, the person was in charge of administration. With the many aspects of administration… I mean, even people with a formal background in health do not always have the capacity of health care administration.”

	Regulation	Inconsistencies in central government regulations	“For example, if some use ‘Pustu Prima’ as the nomenclature for village-level facilities and others do not, I think that is a bit tricky because there will be two regulations to comply with. One is the Minister of Health Regulation Number 43, and the other is the technical guidelines for the ILP itself. Because the contents [of the two regulatory products] are different, it is a bit tricky to have two [regulations]. In my opinion, having one is convenient, so we can handle everything, and the reporting is also easy.”
		Lack of cross-ministerial regulation concerning UPKDK	“If possible, the status of the [ILP] regulation should be elevated from the Minister of Health's decision to a joint regulation of three ministers: the Minister of Health, the Minister of Home Affairs, and the Minister of Villages. When it becomes a [joint] regulation of three ministers, it will be strong [to regulate UPKDK].”
		Technical guideline considered lengthy and unclear	“There are still some parts of the technical guidelines that I find confusing, like cluster 4, which is still unclear. Does Cluster 4 include disease [management]? In my opinion, cluster 4 [environmental health and surveillance] is still confusing.”
	Financing	Lack of financial flexibility	“It is case by case, Sir. For example, for activities such as case follow-up, if there are no cases, we cannot reallocate the money, Sir.”
		UPKDK and *Posyandu* remained under resourced	“The most urgent thing [to support is the facilities. Obviously, [we need] the computer… the Wi-Fi first, Wi-Fi. In the past, actually, the DHO paid IDR 200,000 per month [for internet]. Now, we use Wi-Fi vouchers to be reimbursed. If there is no official proof, it is complicated, so they cannot reimburse it, and it becomes personal costs. So, the average village clinic here uses its own laptop for reporting. Well, in this village, our village clinic has its own. There are some [village clinics who received a laptop] from the village authorities, but only a few.”
	Physical Resources	Challenges in pharmaceutical management at the UPKDK level	“In fact, when we take medication for the village clinics from the pharmacy warehouse, the e-Pus report will record a reduction in the medication from the *Puskesmas*. That is still a problem. But, next year, medication usage in the village clinics will have its separate documentation in the e-Pustu [a designated information system for village clinics].”

		Lack of clear guidance on pharmaceutical supplies at the UPKDK level	“Among us, the pharmacy staff, there is confusion about which medication can be supplied [to village clinics] since currently there is no clear regulation about it until now.”
		Limited medical equipment and consumables at the UPKDK level	“There are not any issues regarding medical devices, because they were all provided in January. But, among the blood sugar, cholesterol, and uric acid testing strips, only blood sugar ones are sufficient. The others are more limited. For example, in terms of cholesterol, we still measure its level when patients have high blood pressure, even though their blood sugar level is normal. But we only have 10 cholesterol strips, whereas for blood sugar, we have 25. We hope that more cholesterol and uric acid strips will be provided so that we encounter patients that meet the eligibility, we can measure them right away.”
		Infrastructure issues across all levels of providers	“The dental equipment is also available, Ma'am, but there is a problem with the water. Our water is used for the assistants, the equipment for the assistants for dental services, the water comes directly from here, which is estimated to contain a lot of iron or calcium, so we are worried that the dental equipment will only last a short time [because the minerals damage it].”
Perceived Facilitators	Local government commitment	District government issued a decree which instructed all district government agencies to support ILP	“Yes, the regent of KSB is very supportive, Ma'am. As soon as news of the minister's arrival and the ILP came out, everything was... immediately followed up with the issuance of a regent's decree.”
		The district and village governments provided financial support to scale up ILP	“Some [village clinic equipment] are provided by the village. The good thing is that the village has [allocated] 10% [of their budget] for health. For example, in September 2023, they mandated 10% of the village fund [to be allocated for health], so this year, whatever the village clinic needs, praise the Lord, can be realized.”
		Support from village authorities played crucial roles for the implementation of ILP	“Based on our experience in the field, since we often do field visit for policy monitoring, we believe at the end of the day, it’s the village heads [that determine policy implementation]”
	Support from community health workers	*Posyandu* CHWs bridged communication between community and *Puskesmas* as well as UPKDK	“The CHWs at *Posyandu* and village clinics work well together. Whatever we [village clinic health workers] ask them to do, household visit for instance; they will work together. All of us here collaborate well.”

		Posyandu CHWs collaborated with Agen Gotong Royong (AGR) at community level	"In terms of household visits, it depends whether it is babies or elderly people. Before the *Posyandu* starts, we will visit their houses with AGR. If they do not show up on the day of *Posyandu*, the AGRs will come to their houses to remind them and pick them up."
	Support from the Ministry of Health	MOH provided technical guidance for implementation of ILP	"Per last November, the legacy of technical assistance from the Ministry of Health was only a pocketbook. During the assistance period, the Ministry of Health representative remained with us for 3 months."
		Non-pilot facilities did not experience the technical support from MOH	"No, we did not receive [any guidance for household visit form from the MOH]. The DHO provided us."
