# Supplementary material for: Barriers and enablers in implementing integrated primary health care: A qualitative assessment in West Sumbawa District, Indonesia
Source: PLOS Glob Public Health. 2026 Sep 11;6(9):e0007238. doi: 10.1371/journal.pgph.0007238 (PMC13567773; doi:10.1371/journal.pgph.0007238)
Supplement: Supplementary file 3 [file pgph.0007238.s005.pdf]

Mentari Widiastuti
Center for Health Policy and Management
Faculty of Medicine, Public Health, and Nursing
Universitas Gadjah Mada
Jalan Medika, Jl. Medika, Sendowo, Sinduadi, Mlati
Sleman, Daerah Istimewa Yogyakarta 55281, Indonesia

10 August 2026

Dear Shailendra Prasad, MD, MPH
Academic Editor
PLOS Global Public Health

Thank you for considering our manuscript entitled "Barriers and enablers in implementing integrated primary health care: a qualitative assessment in West Sumbawa District, Indonesia" for publication in PLOS Global Public Health and for providing us with the reviewers' constructive feedback. We appreciate the time and effort the editorial team and reviewers dedicated to evaluating our work.

We have carefully reviewed all comments and revised the manuscript accordingly. We believe these changes have significantly strengthened the paper. As an attachment to this letter, we provide a point-by-point response detailing how each comment was addressed. For your convenience, we also submitted a marked-up copy of our manuscript and an unmarked version without track changes.

Once again, we thank you for your time and consideration.

Sincerely,

Mentari Widiastuti

Point-by-Point Responses to Viewers

Manuscript Title: Barriers and enablers in implementing integrated primary health care: a qualitative assessment in West Sumbawa District, Indonesia

Submission ID: PGPH-D-26-01877

Editor

Comment 1

"As the corresponding author, your ORCID iD is verified in the submission system and will appear in the published article. PLOS supports the use of ORCID, and we encourage all coauthors to register for an ORCID iD and use it as well. Please encourage your coauthors to verify their ORCID iD within the submission system before final acceptance, as unverified ORCID iDs will not appear in the published article. Only the individual author can complete the verification step; PLOS staff cannot verify ORCID iDs on behalf of authors."

Response:

Thank you for the kind reminder. All co-authors have been notified about this and verified their ORCID iD before final acceptance.

Comment 2

"We note that your Data Availability Statement is currently as follows: All relevant data are provided in the paper and the Supporting Information. Relevant excerpts are also included in the file to describe key themes. Due to the professional nature of the participants and the ethics requirement from the Faculty of Medicine, Public Health, and Nursing Universitas Gadjah Mada, qualitative transcripts cannot be made available publicly. Data are available upon request from the Center for Health Policy and Management, Faculty of Medicine, Public Health, and Nursing Universitas Gadjah Mada Indonesia (chpm@ugm.ac.id) for researchers who meet criteria for access to confidential data, subject to ethical considerations.

Please confirm at this time whether or not your submission contains all raw data required to replicate the results of your study. Authors must share the "minimal data set" for their submission. PLOS defines the minimal data set to consist of the data required to replicate all study findings reported in the article, as well as related metadata and methods (https://journals.plos.org/plosone/s/data-availability#loc-minimal-data-set-definition).

For example, authors should submit the following data:

- The values behind the means, standard deviations and other measures reported;
- The values used to build graphs;
- The points extracted from images for analysis.

Authors do not need to submit their entire data set if only a portion of the data was used in the reported study.

If your submission does not contain these data, please either upload them as Supporting Information files or deposit them to a stable, public repository and provide us with the relevant URLs, DOIs, or accession numbers. For a list of recommended repositories, please see https://journals.plos.org/plosone/s/recommended-repositories.

If there are ethical or legal restrictions on sharing a de-identified data set, please explain them in detail (e.g., data contain potentially sensitive information, data are owned by a third-party organization, etc.) and who has imposed them (e.g., an ethics committee). Please also provide contact information for a data access committee, ethics committee, or other institutional body to which data requests may be sent. If data are owned by a third party, please indicate how others may request data access."

Response:

Thank you for the comment. The qualitative interview transcripts are not publicly available because they contained potential identifying information and participants did not consent to unrestricted public sharing. Although direct identifiers can be removed, the combination of participants' professional roles, health facility characteristics, and contextual information may allow re-identification. Relevant excerpts are also included in the supplementary file to describe key themes. These restrictions were imposed by the Human Research Ethics Committee of the Health Research Ethics Committee of the Faculty of Medicine, Public Health and Nursing, Universitas Gadjah Mada, which approved the study. De-identified data may be made available to qualified researchers for legitimate research purposes following review and approval by the relevant ethics committees and execution of an appropriate data-sharing agreement, where required. Requests for access to the data should be directed to the corresponding author (mentari.widiastuti@mail.ugm.ac.id) in the first instance, who will coordinate review with the relevant institutional ethics committees. We have revised our Data Availability statement in the submission platform.

Comment 3

"Please ensure that your Ethics Statement is available in its entirety at the beginning of your Methods section, under a subheading 'Ethics Statement'."

Response:

Thank you for bringing this into our attention. We have moved our ethics statement to the beginning of the Methods Section, under a subheading 'Ethics Statement' (lines 122-128 in the revised manuscript without track changes).

"Ethics statement

The study received ethics approval from the Internal Review Board of the Faculty of Medicine, Public Health, and Nursing at Universitas Gadjah Mada, Indonesia (Approval Number: KE/FK/1206/EC/2023, KE/FK/1322/EC/2024, KE/FK/1275/EC/2025). Participants provided formal written consent, both in printed and digital formats, for each online or face-to-face data collection. Personally identifiable information was removed from the transcripts to safeguard participants' anonymity and confidentiality. Data privacy measures were implemented."

<u>Comment 4 (Figure Resubmission)</u>

"While revising your submission, we strongly recommend that you use PLOS's NAAS tool (https://ngplosjournals.pagemajik.ai/artanalysis) to test your figure files. NAAS can convert your figure files to the TIFF file type and meet basic requirements (such as print size, resolution), or provide you with a report on issues that do not meet our requirements and that NAAS cannot fix.

After uploading your figures to PLOS's NAAS tool - https://ngplosjournals.pagemajik.ai/artanalysis, NAAS will process the files provided and display the results in the "Uploaded Files" section of the page as the processing is complete. If the uploaded figures meet our requirements (or NAAS is able to fix the files to meet our requirements), the figure will be marked as "fixed" above. If NAAS is unable to fix the files, a red "failed" label will appear above. When NAAS has confirmed that the figure files meet our requirements, please download the file via the download option, and include these NAAS processed figure files when submitting your revised manuscript."

Response:

We thank the reviewer for this helpful suggestion. We have refined our figures using the suggested platform. The figures have been uploaded along with the revised manuscript.

Reviewer 1

<u>Comment 1a</u>

"Dear Editor and Authors,

Thank you for the opportunity to review this highly commendable, rigorous, and timely manuscript. This study provides an invaluable critical analysis of the rollout of the "Integrasi Pelayanan Kesehatan Primer" (ILP) framework in Indonesia under a decentralized public health governance structure. Using a progressive, longitudinal qualitative design (2023–2025) across five distinct time points yields exceptionally rich data that is rarely captured in health system implementation research.

To ensure the manuscript meets the highest standards of methodological transparency and theoretical depth required by PLOS Global Public Health, I would ask the authors to address the following revisions:

1. Strengthening Methodology (Alignment with Qualitative Standards)

a) Data Saturation: With a large sample of 138 participants across 5 sequential rounds, the exact point of structural or thematic data saturation remains vague. Please explicitly clarify in the Data Analysis section during which round or within which participant cluster no new themes emerged, and detail the specific methodological steps taken to verify this saturation."

Response:

Thank you for the positive assessment and the kind suggestion. We acknowledge the reviewer's concern regarding our methods to determine data saturation. Saturation was determined through continuous examination of generated themes across all data collection rounds by the first, second, and third authors. No new themes emerged in the final round of data collection (May 2025). We have addressed this information on lines 279-281 in the unmarked version of the revised manuscript.

“To verify saturation, the first, second, and third authors continuously examined themes generated across all data collection rounds. In the final round (May 2025), no novel themes were generated and the data fit within the existing thematic structure.”

Comment 1b

“Recruitment Strategy & Bias: Under the Sampling sub-section, please clarify how participants were initially approached (e.g., via formal institutional letters via DHO or personal networks). This transparency helps evaluate potential social desirability bias, particularly whether grassroots actors felt monitored, which might skew feedback toward compliance-driven answers.”

Response:

Thank you for the suggestion to clarify our recruitment strategy. In response to the reviewer’s comment, we sent institutional letters through the DHO to formally invite government officials, healthcare providers, and community health workers to participate in the study since having institutional endorsement is considered culturally-appropriate for participant recruitment in the study site. This revision can be found on lines 193-195 in the unmarked version of the revised manuscript.

“Since having institutional endorsement is considered culturally appropriate for participant recruitment in the study site, we also distributed institutional letters through the DHO to formally invite government officials, healthcare providers, and CHWs to participate in the study.”

Comment 1c

“Transcript Verification: Clarify whether feedback obtained from routine validation meetings with the District Health Office (DHO) altered or overrode the raw thematic interpretations derived from the grassroots interviews (cadres and village health workers), ensuring the independent voice of frontline actors is preserved”

Response:

Thank you for the opportunity to clarify about the roles of DHO comments in our analysis. We have addressed this comment on lines 245-248 in the unmarked version of our revised manuscript accordingly.

“Comments and feedback from the DHO were used to contextualize our findings but did not influence the thematic analysis process. The development of final themes remained grounded in the perspective of participants to preserve the independence and authenticity of their voices.”

Comment 2a

“Clinical Competency Boundaries at UPKDK: A striking finding is that some village-level midwives/nurses dispensed antibiotics (Amoxicillin) without a prescription due to regulatory confusion regarding storage guidelines. Please expand upon this in the Discussion section, highlighting its systemic implications for patient safety and Antimicrobial Resistance (AMR), and provide clear recommendations for DHO containment protocols”

Response:

We thank the reviewer for the comment. This is indeed a very important observation and the practice can potentially introduce UPKDK clinics as a new source of inappropriate antibiotic distribution if the regulatory confusion persists. We have addressed this in the unmarked version of our revised manuscript on lines 899-909.

"Notably, gaps in human resource competence were observed in pilot and non-pilot UPKDK clinics, particularly regarding antibiotic dispensing practices. The persistence of this issue in the pilot facility despite their earlier exposure to ILP training, supervision, and implementation support indicate that capacity-building alone is not sufficient to address this challenge. The dispensing of antibiotics without a physician's prescription was reported across pilot and non-pilot UPKDKs, largely attributed to unclear guidelines about pharmaceutical storage and dispensing at village-level facilities. This practice raises patient safety concerns, as inappropriate antibiotic consumption leads to the development of antimicrobial resistance (AMR) [46]. Although community pharmacies have been identified as the primary site of non-prescription antibiotic dispensing in Indonesia [47,48], our finding suggests that UPKDK clinics may emerge as a novel source of inappropriate antibiotic distribution if this operational ambiguity persists."

Comment 2b

"Digital Tool Validation Distrust: Frontline workers deliberately "deducted 20 points" from digital blood pressure readings due to a profound distrust compared to manual tools. Given that digitalization (e.g., the ASIK mobile app) is a core structural pillar of ILP, please address whether this stems from a lack of equipment calibration training and how it affects data integrity in the wider information system reform."

Response:

Thank you for the comment. We agree this issue can undermine data quality in the wider information system. To the extent of our observation, this issue stems from the lack of training, standardized protocols, and technical support from DHO when introducing new technologies to rural health workers, since a follow-up response from the participant describes the lack of response and follow-up actions from the DHO despite the participant's attempt to raise the issue. We have addressed this in the unmarked version of our revised manuscript on lines 910-918.

"We also identified an issue with the introduction of medical technologies at the UPKDK level. A participant from a non-pilot UPKDK clinic reported difficulty in using a newly introduced digital blood pressure measurement tool supplied by the DHO. The device was perceived to consistently produce higher readings than the old one. As such, blood pressure values were sometimes adjusted downward by 20 mmHg based on the health worker's judgment. This example highlights insufficiency in training, standardized measurement protocols, device validation mechanisms, and technical support for rural health workers when new technology is introduced. Such gaps may compromise not only treatment accuracy, but also data quality in the information system, which is essential to support structural integration [7]."

Comment 3a

"The integration frameworks of Shortell et al. (clinical/fiscal accountability) and Singer et al. (structural, functional, normative, etc.) introduced in the Introduction should be explicitly tied back

into the empirical findings within the Discussion section (e.g., mapping village fund blockages to functional/fiscal integration barriers)."

Response:

Thank you for the suggestion to link our findings with our theoretical frameworks in the Discussion. We have addressed this on the following lines.

Lines 883-887

"On top of that, the issuance of district free health care policy hindered Puskesmas from increasing their revenue to equitably fund promotive-preventive and curative services, despite having the flexible financial autonomy status. Drawing on the integration model by Singer et al. [7], these findings provided an example of issues in the functional aspect of integration."

Lines 914-918

"This example highlights insufficiency in training, standardized measurement protocols, device validation mechanisms, and technical support for rural health workers when new technology is introduced. Such gaps may compromise not only treatment accuracy, but also data quality in the information system, which is essential to support structural integration [7]."

Lines 919-923

"The variable, yet fragmented and not interoperable digital applications for data management and reporting, as reported by frontline health worker participants, further created complexity in establishing an integrated information system. Singer et al. emphasize that the lack of interoperable information systems may undermine process integration as it hinders information sharing [7]."

Lines 943-957

"Our findings suggested the importance of redefining "integration" within the IPHC concept. Drawing from the lessons of WSD, the implementation of IPHC in Indonesia through the ILP approach was still heavily focused on the administrative and input aspects, with few emphases on the social and process aspect of integration. Shortell et al. argued that effective integration requires not only operational levers, such as financing and information system, but also personnel that share organizational missions and values, as well as strategic planning and continuous improvement processes across the entire system [6]. Singer et al. further suggested that interpersonal integration among those involved in patient care, including the patients themselves, is pivotal to achieving care outcomes, such as efficiency and satisfaction [7]. In summary, integration requires coordinated human resources, functions, activities, and operating units [6,7] and, thus, should not be viewed as the end state, but rather as a process to achieve outcomes. These outcomes may include improved care coordination, continuity, quality, efficiency, accountability, and better patient experiences [7]. This understanding needs to be further translated into a clear theory of change encompassing the structure, input, process, output, and outcomes to facilitate various stakeholders understand, implement, and measure ILP."

<u>Comment 3b</u>

"Contextualization of National Screening Policies: Please clarify whether West Sumbawa District has issued localized regulations or unique SOPs to harmonize Kemenkes' 39 ILP screening items with the

newly introduced presidential comprehensive national free health screening package (noted in Round 5, May 2025)."

Response:

We thank the reviewer for the important question. We have clarified this in the Results (lines 628-639) and Discussion (lines 981-984) sections of the unmarked manuscript file.

Lines 628-639
Gaps in local translation of national policies were also evident in the implementation of screening initiatives. By the final round of data collection (May 2025), there were no accounts regarding district regulations or guidelines that formally harmonized the ILP screening packages with the national screening initiative. Health workers also raised concerns regarding activity duplication and the lack of coordination between the two initiatives.

"There are a lot of components [in the new screening initiative]. The program also comes with a new application [to record screening data]. It is unfortunate that they introduce a new screening program, whereas the current [ILP] one has not been implemented well. I find it confusing how these two initiatives can be synchronized."

(Health worker, Non-pilot UPKDK 05, May 2025)

Lines 981-984
"The absence of local guidelines for ILP and regulations harmonizing ILP screening packages with the new national screening program suggests that future policies should provide subnational governments with clear operational pathways for integrating overlapping programs and initiatives."

Comment 3c

"Please insert a deeper comparative analysis regarding the pilot vs. non-pilot dynamics here. While your data beautifully captures both settings, the discussion should explicitly leverage this contrast to elaborate on 'scaling-up' challenges in decentralized settings. Specifically, address:
- Acceptability Dimensions: Did non-pilot sites display implicit policy resistance, or was slower adoption purely driven by operational frustration with the dense, non-actionable MOH guidelines?
- Readiness and Infrastructure Variance: Elaborate on how physical resource deficits (e.g., test strips, blackouts, lack of water) varied, highlighting the structural inequities inherent when centralized top-down funding injections are withdrawn during rapid scale-ups."

Response:

Thank you for this important suggestion. Upon further review of the data, we observed that scale-up challenges may stem less from policy resistance and more from operational frustration. We address this on lines 856-861.

"However, slower adoption of ILP was observed in non-pilot facilities, where participants cited the lack of practical supervision and the lengthy, information-dense national guidelines as reasons for this. Therefore, challenges in non-pilot facilities do not reflect participants' resistance to ILP but are primarily attributed to technical and operational setbacks. Nonetheless, by the end of data collection, all Puskesmas *already implemented ILP, although to different extents."*

In terms of physical resources, our findings did not indicate inequities between pilot and non-pilot facilities. Although pilot facilities received medical devices earlier, both groups were ultimately equipped by the DHO to support ILP delivery. Furthermore, infrastructure issues such as shortages of test strips, electricity disruptions, and unreliable water supply were reported across all facilities regardless of pilot status. These findings suggest that variation in ILP adoption was more closely related to discrepancies in learning, capacity-building, and implementation support than with resource availability. We clarified this point on lines 926-932.

"Challenges related to physical resources, pharmaceutical supply and equipment, as well as water and electricity were identified at all primary care provider levels regardless of the pilot status. The persistence of these constraints in pilot and non-pilot facilities further indicates that barriers in ILP scale-up are more closely related to discrepancies in learning and capacity-building support than with resource availability. Overall, our results suggested that establishing IPHC requires coordinating public health functions, which is aligned with the principles of an effective model of care introduced by the World Health Organization and the United Nations Children's Fund [3]."

Therefore, we also emphasized that IPHC requires system-level reforms in "Recommendations for Policy, Practice, and Future Research" on lines 993-996.

"Fourth, effective scale-up of IPHC requires broader health system strengthening [54,55]. The reform should simultaneously address strategic and operational building blocks to create an enabling system for governments, providers, and societies for establishing equitable, comprehensive, and integrated health services [3]."

Reviewer 2

General Comment

"Thank you for the opportunity to review this paper on an important program, that is some ways can be viewed as a global exemplar in PHC.

The manuscript does provide a valuable implementation analysis of ILP in a decentralized Indonesian context. The study provides rich perspectives from implementers and managers and also identifies important barriers (HR, financing, governance, information systems) and facilitators (government support, CHWs, MOH leadership). It is informative and provides a grounded perspective that is useful to PHC scholars and administrators.

Its major limitations are that patient perspectives, patient journey analysis, assessment of whether ILP improves continuity, trust, care coordination, or population outcomes etc are not included.

Considerations for strengthening the paper:

The paper will be strengthened by moving from a predominantly implementation-science framing toward a more explanatory health systems perspective focused on continuity, accountability, and stewardship as primary functions of interest. Explicitly discussing these will be valuable because integration is a mechanism towards the end (performance, accountability, and outcomes), and not an end in and of itself.

1) primarily descriptive and implementation-focused. The paper provides only limited explanation of the deeper system mechanisms generating these challenges.

- The paper would benefit from a stronger theoretical framing that moves beyond barriers and facilitators toward understanding how integrated primary healthcare functions as a system.

2) The study does not include any patient or community experience. Additionally, it does not examine any outcomes or performance of core processes/functions of PHC such as Continuity, Coordination, Comprehensiveness, First contact care etc.

- Discussion of how integration is (or even intended to) influence accountability and the experience for patients across the entire care journey will be useful."

Response:

We thank the reviewer for highlighting the importance of continuity, accountability, stewardship, patient experience, and care journey analysis in the evaluation of integrated primary health care. We agree that integration should be viewed as a means to achieve broader health system objectives rather than an end in itself.

However, the objective of the present study was intentionally focused on the implementation of ILP at the district level and the health system factors influencing its rollout. The study was designed as an implementation-focused qualitative assessment, drawing on integration frameworks from Singer et al. and Shortell et al. to understand implementation processes and systemic determinants rather than patient-level outcomes or performance measures.

While we appreciate the value of alternative theoretical approaches focused on stewardship, accountability, and continuity, we have retained the original conceptual framing because it aligns with the study's predefined aims, design, data collection strategy, and analytical focus on implementation.

In response to the reviewer's suggestion, we have revised the Discussion to more explicitly acknowledge that integration is intended to contribute to broader outcomes such as continuity, coordination, accountability, and patient experience (lines 951-957).

"In summary, integration requires coordinated human resources, functions, activities, and operating units [6,7] and, thus, should not be viewed as the end state, but rather as a process to achieve outcomes. These outcomes may include improved care coordination, continuity, quality, efficiency, accountability, and better patient experiences [7]. This understanding needs to be further translated into a clear theory of change encompassing the structure, input, process, output, and outcomes to facilitate various stakeholders understand, implement, and measure ILP."

We have also added text to the Limitations section to clarify that examining these outcomes was beyond the scope of the current study and should be explored in future research (lines 968-973 and lines 1000-1002).

Lines 968-973

"The repeated data collection also helped us build rapport with our participants. Third, the study is conducted in Indonesia and focused on the health system factors shaping ILP implementation at the district level rather than the effect of ILP on patient, service, and population outcomes. Consequently, while outcomes such as continuity of care, coordination, comprehensiveness of care, accountability, or

service delivery stewardship are considered important aspects of IPHC, they were beyond the scope of this paper."

Lines 1000-1002
"Our study also highlights several priorities for future research. We recommend examinations of how IPHC contributes to better service integration, health system performance, and population outcomes."

Reviewer 3

<u>Comment 1</u>

"Thank you for the opportunity to review this manuscript. The authors present a timely and relevant qualitative evaluation of a complex health service intervention. The study captures stakeholder perspectives from both pilot and non-pilot sites to understand implementation barriers and system readiness. Notably, the study was conducted during a period of significant health service transition in the aftermath of the pandemic, which adds valuable real-world context to the findings.

The paper is well-written, demonstrates strong local buy-in, and has a robust qualitative design. However, there are a few areas where the methodological description and the discussion's structure could be tightened to improve clarity for a wider audience.

1. *Framework nomenclature and clarity*

The authors state that they are utilising the complex intervention framework by Skivington et al. (2021). While this is accurate, international readers and policy makers more readily recognise this methodology by its institutional name. I recommend that upon first mention, the authors explicitly introduce it as the updated UK Medical Research Council (MRC) / National Institute for Health and Care Research (NIHR) Framework for Complex Interventions (Skivington et al., 2021). This will clarify the study design and give the paper immediate structural authority."

Response:

Thank you for bringing this into our attention. We have revised this accordingly in the unmarked version of our revised manuscript on lines 266-268.

"These themes were developed based on the updated United Kingdom Medical Research Council (MRC)/National Institute for Health and Care Research (NIHR) Framework for Complex Interventions."

<u>Comment 2</u>

"2. Specification of the qualitative methodology

While the manuscript focuses heavily on capturing the participants' lived experiences, perceptions, and immediate practical realities, the precise qualitative methodology or school of thought guiding the analysis remains unstated. The approach appears to align closely with descriptive phenomenology, given the focus on documenting these experiences as they occurred on the ground. Recommendation: The authors should explicitly name and justify the study design and their qualitative approach in the Methods section. If descriptive phenomenology was indeed the guiding methodology, stating this clearly will help the reader understand how the team managed researcher bias (e.g., through bracketing) and

how they arrived at their thematic structure. The inclusion of the reflexivity/positionality statements is useful, but could be expanded."

Response:

Thank you for the opportunity to clarify on our qualitative design. While descriptive phenomenology may have relevance to our approach, our study used a semantic thematic analysis with a realist paradigm. Detailed information on our study paradigm and designs is provided on lines 131-135 and 251-253 in the unmarked version of our revised manuscript.

Lines 131-135

"The study employed a qualitative design using thematic analysis with a realist paradigm. As described by Braun and Clarke, a realist paradigm allows a straightforward interpretation of meaning based on participants' statements [26]. The study was performed and reported in accordance with the Consolidated Criteria for Reporting Qualitative Research (COREQ) framework [27] (see S1 File for details)."

Lines 251-253

"The analysis was conducted using a semantic approach, with the interpretation of broader meanings and implications directly based on participants' description of their views and experiences [26]."

<u>Comment 2</u>

"3. Sample distribution and analysis strategy

There is a distinct imbalance in the qualitative sample, with the number of interviewees from the non-pilot sites heavily outweighing those from the single active pilot site. This is a common challenge in real-world evaluations, but it alters the nature of the data. Respondents from the active pilot site provide data on actual implementation (lived experience), while non-pilot participants discuss anticipated barriers (hypothetical context).

To ensure that the volume of non-pilot data does not dilute the rich, ground-level micro-processes from the pilot site, the authors should clarify their analytical strategy. Please clarify in the Methods section whether the transcripts were pooled or analysed as two distinct sub-datasets (e.g., a process case study for the pilot site vs a readiness assessment for the non-pilot sites). Acknowledging this sample dynamic in the Discussion would also enhance the study's transparency."

Response:

Thank you for this insightful observation. We acknowledge the reviewer's concern about our sample distribution and analysis strategy. We have provided information about this on lines 256-264 in the unmarked version of our revised manuscript.

"During initial coding, all transcripts were coded using a shared codebook and labelled with pilot and non-pilot. Coded data were then compared across pilot and non-pilot PHCs during interpretation. During theme development, transcripts from both groups were analyzed together to identify themes that reflected shared meanings across implementation contexts. Although the number of pilot and non-pilot facilities was not equal, non-pilot facilities had been gradually exposed to ILP implementation throughout the data collection period. As such, participants' accounts reflected different stages of ILP

implementation rather than entirely distinct contexts of pilot and non-pilot facilities. Themes were developed based on the interpretation of transcripts using a semantic approach."

Additionally, we have acknowledged this implementation variation in the Discussion (lines 860-861) to assist readers in interpreting the findings.

"Nonetheless, by the end of data collection, all Puskesmas *already implemented ILP, although to different extents."*

Comment 4

"4. Discussion section structure
The transition into the Discussion section currently feels a bit abrupt. The opening paragraph would benefit from a high-level summary that brings the reader back to the study's core elements. I suggest restructuring the first paragraph of the Discussion to clearly and concisely restate:

** The overarching aim and objectives of the evaluation.*
** The chosen study design and framework.*
** A high-level synthesis of the key findings.*
This will provide a useful road map before diving into the specific thematic interpretations."

Response:

We thank the reviewer for the suggestion. We have added a high-level summary in the beginning of the Discussion as suggested. Specifically, it has been provided on lines 836-850.

"This study examined the implementation of IPHC through the ILP approach in Indonesia, a country with a complex and decentralized health system. The study was conducted in a district designated as a national pilot ILP site. We used a qualitative, thematic analysis design and employed a realist paradigm, whereby meaning was grounded on participants' accounts [26]. Our understanding of service integration was guided by two complementary models. Shortell et al. conceptualize integration as physician-system, functional, and clinical, with clinical integration considered the most pivotal for healthcare system performance [6]. Singer et al. expands this framework by identifying structural, functional, normative, interpersonal, and process dimensions of integration [7]. Together, these models inform that a successful service integration requires the alignment of human resources, organizational infrastructures, and culture, alongside effective collaboration and coordination. In addition, we adapted the MRC/NIHR Framework for Complex Intervention Evaluation [41] to define key elements of intervention implementation and to guide thematic development. Our findings highlight the importance of structural and operational redesign, as well as a deep understanding of local social, political, and organizational contexts for establishing IPHC in a decentralized system."

Comment 5

"5. Separation of strengths, limitations, and recommendations
Towards the end of the Discussion, the authors weave the study's strengths and limitations directly into the recommendations. This blending can obscure the distinct value of both components. I recommend separating these into clear, dedicated subsections with explicit subheadings (e.g., "Strengths and Limitations" followed by "Recommendations for Practice and Policy"). This change will make the actionable insights stand out far more sharply for readers and decision-makers."

Response:

We thank the reviewer for this important feedback. We have added the headings as suggested on lines 959 and 978. As for Recommendations, we wrote it as "Recommendations for Future Practice, Policy, and Research" since the section contains suggestions about potential research topics and designs drawing from our findings and current limitations.
