# Supplementary material for: Barriers and enablers in implementing integrated primary health care: A qualitative assessment in West Sumbawa District, Indonesia
Source: PLOS Glob Public Health. 2026 Sep 11;6(9):e0007238. doi: 10.1371/journal.pgph.0007238 (PMC13567773; doi:10.1371/journal.pgph.0007238)
Supplement: Peer review history

# Peer review: Decision Letter 0

**DOI:** 10.1371/journal.pgph.0007238.r001

**Authors:** Shailendra Prasad

28 Jun 2026

PGPH-D-26-01877

Barriers and enablers in implementing integrated primary health care: a qualitative assessment in West Sumbawa District, Indonesia

PLOS Global Public Health

Dear Dr. Widiastuti,

Thank you for submitting your manuscript to PLOS Global Public Health. After careful consideration, we feel that it has merit but does not fully meet PLOS Global Public Health’s publication criteria as it currently stands. Therefore, we invite you to submit a revised version of the manuscript that addresses the points raised during the review process.

Please submit your revised manuscript by Aug 27 2026 11:59PM. If you will need more time than this to complete your revisions, please reply to this message or contact the journal office at globalpubhealth@plos.org. When you're ready to submit your revision, log on to https://www.editorialmanager.com/pgph/ and select the 'Submissions Needing Revision' folder to locate your manuscript file.

Please include the following items when submitting your revised manuscript:

- A letter that responds to each point raised by the editor and reviewer(s). You should upload this letter as a separate file labeled 'Response to Reviewers'.
- A marked-up copy of your manuscript that highlights changes made to the original version. You should upload this as a separate file labeled 'Revised Manuscript with Track Changes'.
- An unmarked version of your revised paper without tracked changes. You should upload this as a separate file labeled 'Manuscript'.

Guidelines for resubmitting your figure files are available below the reviewer comments at the end of this letter.

As the corresponding author, your ORCID iD is verified in the submission system and will appear in the published article. PLOS supports the use of ORCID, and we encourage all coauthors to register for an ORCID iD and use it as well. Please encourage your coauthors to verify their ORCID iD within the submission system before final acceptance, as unverified ORCID iDs will not appear in the published article. *Only*  the individual author can complete the verification step; PLOS staff *cannot*  verify ORCID iDs on behalf of authors.

We look forward to receiving your revised manuscript.

Kind regards,

Shailendra Prasad, MD, MPH

Academic Editor

PLOS Global Public Health

**Journal Requirements:**

1. We note that your Data Availability Statement is currently as follows: All relevant data are provided in the paper and the Supporting Information. Relevant excerpts are also included in the file to describe key themes. Due to the professional nature of the participants and the ethics requirement from the Faculty of Medicine, Public Health, and Nursing Universitas Gadjah Mada, qualitative transcripts cannot be made available publicly. Data are available upon request from the Center for Health Policy and Management, Faculty of Medicine, Public Health, and Nursing Universitas Gadjah Mada Indonesia (chpm@ugm.ac.id) for researchers who meet criteria for access to confidential data, subject to ethical considerations.

Please confirm at this time whether or not your submission contains all raw data required to replicate the results of your study. Authors must share the “minimal data set” for their submission. PLOS defines the minimal data set to consist of the data required to replicate all study findings reported in the article, as well as related metadata and methods (https://journals.plos.org/plosone/s/data-availability#loc-minimal-data-set-definition).

For example, authors should submit the following data:

- The values behind the means, standard deviations and other measures reported;

- The values used to build graphs;

- The points extracted from images for analysis.

Authors do not need to submit their entire data set if only a portion of the data was used in the reported study.

If your submission does not contain these data, please either upload them as Supporting Information files or deposit them to a stable, public repository and provide us with the relevant URLs, DOIs, or accession numbers. For a list of recommended repositories, please see https://journals.plos.org/plosone/s/recommended-repositories.

If there are ethical or legal restrictions on sharing a de-identified data set, please explain them in detail (e.g., data contain potentially sensitive information, data are owned by a third-party organization, etc.) and who has imposed them (e.g., an ethics committee). Please also provide contact information for a data access committee, ethics committee, or other institutional body to which data requests may be sent. If data are owned by a third party, please indicate how others may request data access.

2. Please ensure that your Ethics Statement is available in its entirety at the beginning of your Methods section, under a subheading 'Ethics Statement'.

If the reviewer comments include a recommendation to cite specific previously published works, please review and evaluate these publications to determine whether they are relevant and should be cited. There is no requirement to cite these works unless the editor has indicated otherwise.

**Additional Editor Comments (if provided):**

[Note: HTML markup is below. Please do not edit.]

Reviewers' comments:

Reviewer's Responses to Questions

**Comments to the Author**

1. Does this manuscript meet PLOS Global Public Health’s publication criteria?

Reviewer #1: Partly

Reviewer #2: Yes

Reviewer #3: Yes

**********

2. Has the statistical analysis been performed appropriately and rigorously?

Reviewer #1: N/A

Reviewer #2: Yes

Reviewer #3: N/A

**********

3. Have the authors made all data underlying the findings in their manuscript fully available (please refer to the Data Availability Statement at the start of the manuscript PDF file)?

The PLOS Data policy

Reviewer #1: Yes

Reviewer #2: Yes

Reviewer #3: Yes

**********

4. Is the manuscript presented in an intelligible fashion and written in standard English?

Reviewer #1: Yes

Reviewer #2: Yes

Reviewer #3: Yes

**********

**Reviewer #1:**  Dear Editor and Authors,

Thank you for the opportunity to review this highly commendable, rigorous, and timely manuscript. This study provides an invaluable critical analysis of the rollout of the "Integrasi Pelayanan Kesehatan Primer" (ILP) framework in Indonesia under a decentralized public health governance structure. Using a progressive, longitudinal qualitative design (2023–2025) across five distinct time points yields exceptionally rich data that is rarely captured in health system implementation research.

To ensure the manuscript meets the highest standards of methodological transparency and theoretical depth required by PLOS Global Public Health, I would ask the authors to address the following revisions:

1. Strengthening Methodology (Alignment with Qualitative Standards)

a) Data Saturation: With a large sample of 138 participants across 5 sequential rounds, the exact point of structural or thematic data saturation remains vague. Please explicitly clarify in the Data Analysis section during which round or within which participant cluster no new themes emerged, and detail the specific methodological steps taken to verify this saturation.

b) Recruitment Strategy & Bias: Under the Sampling sub-section, please clarify how participants were initially approached (e.g., via formal institutional letters via DHO or personal networks). This transparency helps evaluate potential social desirability bias, particularly whether grassroots actors felt monitored, which might skew feedback toward compliance-driven answers.

c) Transcript Verification: Clarify whether feedback obtained from routine validation meetings with the District Health Office (DHO) altered or overrode the raw thematic interpretations derived from the grassroots interviews (cadres and village health workers), ensuring the independent voice of frontline actors is preserved.

2. Sharpening Critical Analysis of Frontline Anomalies

a) Clinical Competency Boundaries at UPKDK: A striking finding is that some village-level midwives/nurses dispensed antibiotics (Amoxicillin) without a prescription due to regulatory confusion regarding storage guidelines. Please expand upon this in the Discussion section, highlighting its systemic implications for patient safety and Antimicrobial Resistance (AMR), and provide clear recommendations for DHO containment protocols.

b) Digital Tool Validation Distrust: Frontline workers deliberately "deducted 20 points" from digital blood pressure readings due to a profound distrust compared to manual tools. Given that digitalization (e.g., the ASIK mobile app) is a core structural pillar of ILP, please address whether this stems from a lack of equipment calibration training and how it affects data integrity in the wider information system reform.

3. Theoretical Synchronization and Formatting

a) The integration frameworks of Shortell et al. (clinical/fiscal accountability) and Singer et al. (structural, functional, normative, etc.) introduced in the Introduction should be explicitly tied back into the empirical findings within the Discussion section (e.g., mapping village fund blockages to functional/fiscal integration barriers).

b) Contextualization of National Screening Policies: Please clarify whether West Sumbawa District has issued localized regulations or unique SOPs to harmonize Kemenkes' 39 ILP screening items with the newly introduced presidential comprehensive national free health screening package (noted in Round 5, May 2025).

c) Please insert a deeper comparative analysis regarding the pilot vs. non-pilot dynamics here. While your data beautifully captures both settings, the discussion should explicitly leverage this contrast to elaborate on 'scaling-up' challenges in decentralized settings. Specifically, address:

- Acceptability Dimensions: Did non-pilot sites display implicit policy resistance, or was slower adoption purely driven by operational frustration with the dense, non-actionable MOH guidelines?

- Readiness and Infrastructure Variance: Elaborate on how physical resource deficits (e.g., test strips, blackouts, lack of water) varied, highlighting the structural inequities inherent when centralized top-down funding injections are withdrawn during rapid scale-ups.

Recommendation: Accepted with Minor Revisions.

**Reviewer #2:**  Thank you for the opportunity to review this paper on an important program, that is some ways can be viewed as a global exemplar in PHC.

The manuscript does provide a valuable implementation analysis of ILP in a decentralized Indonesian context. The study provides rich perspectives from implementers and managers and also identifies important barriers (HR, financing, governance, information systems) and facilitators (government support, CHWs, MOH leadership). It is informative and provides a grounded perspective that is useful to PHC scholars and administrators.

Its major limitations are that patient perspectives, patient journey analysis, assessment of whether ILP improves continuity, trust, care coordination, or population outcomes etc are not included.

Considerations for strengthening the paper:

The paper will be strengthened by moving from a predominantly implementation-science framing toward a more explanatory health systems perspective focused on continuity, accountability, and stewardship as primary functions of interest. Explicitly discussing these will be valuable because integration is a mechanism towards the end (performance, accountability, and outcomes), and not an end in and of itself.

1) primarily descriptive and implementation-focused. The paper provides only limited explanation of the deeper system mechanisms generating these challenges.

- The paper would benefit from a stronger theoretical framing that moves beyond barriers and facilitators toward understanding how integrated primary healthcare functions as a system.

2) The study does not include any patient or community experience. Additionally, it does not examine any outcomes or performance of core processes/functions of PHC such as Continuity, Coordination, Comprehensiveness, First contact care etc.

- Discussion of how integration is (or even intended to) influence accountability and the experience for patients across the entire care journey will be useful.

**Reviewer #3:**  Thank you for the opportunity to review this manuscript. The authors present a timely and relevant qualitative evaluation of a complex health service intervention. The study captures stakeholder perspectives from both pilot and non-pilot sites to understand implementation barriers and system readiness. Notably, the study was conducted during a period of significant health service transition in the aftermath of the pandemic, which adds valuable real-world context to the findings.

The paper is well-written, demonstrates strong local buy-in, and has a robust qualitative design. However, there are a few areas where the methodological description and the discussion's structure could be tightened to improve clarity for a wider audience.

1. Framework nomenclature and clarity

The authors state that they are utilising the complex intervention framework by Skivington et al. (2021). While this is accurate, international readers and policy makers more readily recognise this methodology by its institutional name. I recommend that upon first mention, the authors explicitly introduce it as the updated UK Medical Research Council (MRC) / National Institute for Health and Care Research (NIHR) Framework for Complex Interventions (Skivington et al., 2021). This will clarify the study design and give the paper immediate structural authority.

2. Specification of the qualitative methodology

While the manuscript focuses heavily on capturing the participants' lived experiences, perceptions, and immediate practical realities, the precise qualitative methodology or school of thought guiding the analysis remains unstated. The approach appears to align closely with descriptive phenomenology, given the focus on documenting these experiences as they occurred on the ground. Recommendation: The authors should explicitly name and justify the study design and their qualitative approach in the Methods section. If descriptive phenomenology was indeed the guiding methodology, stating this clearly will help the reader understand how the team managed researcher bias (e.g., through bracketing) and how they arrived at their thematic structure. The inclusion of the reflexivity/positionality statements is useful, but could be expanded.

3. Sample distribution and analysis strategy

There is a distinct imbalance in the qualitative sample, with the number of interviewees from the non-pilot sites heavily outweighing those from the single active pilot site. This is a common challenge in real-world evaluations, but it alters the nature of the data. Respondents from the active pilot site provide data on actual implementation (lived experience), while non-pilot participants discuss anticipated barriers (hypothetical context).

To ensure that the volume of non-pilot data does not dilute the rich, ground-level micro-processes from the pilot site, the authors should clarify their analytical strategy. Please clarify in the Methods section whether the transcripts were pooled or analysed as two distinct sub-datasets (e.g., a process case study for the pilot site vs a readiness assessment for the non-pilot sites). Acknowledging this sample dynamic in the Discussion would also enhance the study's transparency.

4. Discussion section structure

The transition into the Discussion section currently feels a bit abrupt. The opening paragraph would benefit from a high-level summary that brings the reader back to the study's core elements. I suggest restructuring the first paragraph of the Discussion to clearly and concisely restate:

* The overarching aim and objectives of the evaluation.

* The chosen study design and framework.

* A high-level synthesis of the key findings.

This will provide a useful road map before diving into the specific thematic interpretations.

5. Separation of strengths, limitations, and recommendations

Towards the end of the Discussion, the authors weave the study's strengths and limitations directly into the recommendations. This blending can obscure the distinct value of both components. I recommend separating these into clear, dedicated subsections with explicit subheadings (e.g., "Strengths and Limitations" followed by "Recommendations for Practice and Policy"). This change will make the actionable insights stand out far more sharply for readers and decision-makers.

**********

what does this mean?). If published, this will include your full peer review and any attached files.

**Do you want your identity to be public for this peer review?**  If you choose “no”, your identity will remain anonymous but your review may still be made public.

For information about this choice, including consent withdrawal, please see our Privacy Policy

Reviewer #1: No

Reviewer #2: **Yes:**  Ramakrishna Prasad

Reviewer #3: No

**********

[NOTE: If reviewer comments were submitted as an attachment file, they will be attached to this email and accessible via the submission site. Please log into your account, locate the manuscript record, and check for the action link "View Attachments". If this link does not appear, there are no attachment files.]

**Figure Resubmissions:**

Attachment Submitted filename: PGPH-D-26-01877- review.docx

# Author comment: Author response to Decision Letter 1

**DOI:** 10.1371/journal.pgph.0007238.r002

11 Aug 2026

Attachment Submitted filename: Widiastuti et al - Response to Reviewers.pdf

# Editor report: Decision Letter 1

**DOI:** 10.1371/journal.pgph.0007238.r003

**Authors:** Shailendra Prasad

18 Aug 2026

Barriers and enablers in implementing integrated primary health care: a qualitative assessment in West Sumbawa District, Indonesia

PGPH-D-26-01877R1

Dear Ms. Widiastuti,

We are pleased to inform you that your manuscript 'Barriers and enablers in implementing integrated primary health care: a qualitative assessment in West Sumbawa District, Indonesia' has been provisionally accepted for publication in PLOS Global Public Health.

Before your manuscript can be formally accepted you will need to complete some formatting changes, which you will receive in a follow up email. A member of our team will be in touch with a set of requests.

Please note that your manuscript will not be scheduled for publication until you have made the required changes, so a swift response is appreciated.

IMPORTANT: The editorial review process is now complete. PLOS will only permit corrections to spelling, formatting or significant scientific errors from this point onwards. Requests for major changes, or any which affect the scientific understanding of your work, will cause delays to the publication date of your manuscript.

If your institution or institutions have a press office, please notify them about your upcoming paper to help maximize its impact. If they'll be preparing press materials, please inform our press team as soon as possible -- no later than 48 hours after receiving the formal acceptance. Your manuscript will remain under strict press embargo until 2 pm Eastern Time on the date of publication. For more information, please contact globalpubhealth@plos.org.

Thank you again for supporting Open Access publishing; we are looking forward to publishing your work in PLOS Global Public Health.

Best regards,

Shailendra Prasad, MD, MPH

Academic Editor

PLOS Global Public Health

***********************************************************

Thank you for your diligence in addressing all the concerns of the peer reviewers. I appreciate the detailed response.

Reviewer Comments (if any, and for reference):
